# Exploring the link between water-soluble vitamins and aging-associated immune system status

**DOI:** 10.3389/fimmu.2026.1771591

**Published:** 2026-05-26

**Authors:** Hendrik Schmieder, Katja Detert, Christian Leischner, Sascha Venturelli, Markus Burkard

**Affiliations:** 1 Department of Nutritional Biochemistry, University of Hohenheim, Stuttgart, Germany; 2 Department of Vegetative and Clinical Physiology, Institute of Physiology, University of Tuebingen, Tuebingen, Germany; 3 Department of Internal Medicine VIII, Medical Oncology and Pneumology, Virotherapy Center Tübingen (VCT), University Hospital Tuebingen, Tuebingen, Germany

**Keywords:** elderly, immune system, immunomodulation, older people, water-soluble vitamins

## Abstract

**Background:**

Aging is associated with numerous physiological changes that contribute to immunosenescence and inflammaging, resulting in a decline in immune defense and an increased susceptibility to infections. Older adults are at risk of inadequate dietary intake and suboptimal supply of micronutrients, including water-soluble vitamins, even those generally considered sufficient in the overall population. These deficiencies may have relevant but often underrecognized consequences for immune function in the elderly.

**Objective:**

This review article aims to provide a well-defined overview and comprehensive assessment of the immunomodulatory properties of water-soluble vitamins in individuals aged 65 and older, with a particular focus on vitamins B1, B2, B3, B5, B6, B7, B9, B12 and C.

**Methods:**

We reviewed and discussed available human-, animal- and *in vitro* studies examining the relationship between water-soluble vitamin status and immune parameters in older adults. In addition, we summarized, compared and graphically illustrated Recommended Dietary Allowances, Nutrient Reference Values and Tolerable Upper Intake Levels for the investigated vitamins across different reference systems.

**Conclusion:**

Aging and insufficient plasma levels of water-soluble vitamins are both associated with immune decline and chronic inflammation. Adequate dietary intake and supplementation of vitamins B1–B12 and vitamin C may help counteract these aging-related immune alterations by supporting immune cell metabolism and reducing susceptibility to infections. Further human studies, and randomized controlled trials in particular, are required to better define the role of water-soluble vitamins in maintaining immune function during aging.

## 1 Introduction

The elderly, a term commonly describing people aged 65 and older but without a uniform definition, represent the fastest growing age group with an estimated medical- and lifestyle-induced life expectancy extension at birth of about five years by 2050 (1–3). Accordingly, aging and aging-related changes comprising telomere attrition, cellular senescence, chronic inflammation and genome instability among others, the so-called “hallmarks of aging” (4), accompanied by prominent immunologic alterations are becoming increasingly relevant. “Immunosenescence” describes the aging-associated gradual deterioration of the innate- and adaptive immune system, accompanied by a chronic state of low-grade inflammation which ultimately contributes to poor vaccination efficacy. Functional impairment and characteristic alterations of various immune cell populations including B cells and T cells, lead to an increased susceptibility toward pathogens and therefore infectious diseases. These factors result in accumulation of age-related disorders and a compromised health overall (5–7). More specifically, the reduced number of T- or B-lymphocytes and related subpopulations as well as their altered antibody production, antigen presentation and cytokine secretion (favoring Th2 T-helper responses while repressing Th1 responses), along with a reduction of primary (bone marrow and thymus) and peripheral lymphoid tissues account for elevated incidents of, e.g., bronchitis or similar secondary bacterial respiratory tract infections. These are accompanied by an increase in morbidity and mortality due to catching the common cold (7–14). To further elucidate the topic of immunosenescence and inflammaging, Oh and colleagues comprehensively summarized and reviewed the key immunologic changes that most persons experience during the process of aging, including reduced phagocytic activities of macrophages, altered memory B cell homeostasis and increased numbers of senescent or exhausted T cells as well as limited diversity in B- and T-cell receptor repertoire, just to name a few (5).

**Figure 1:**
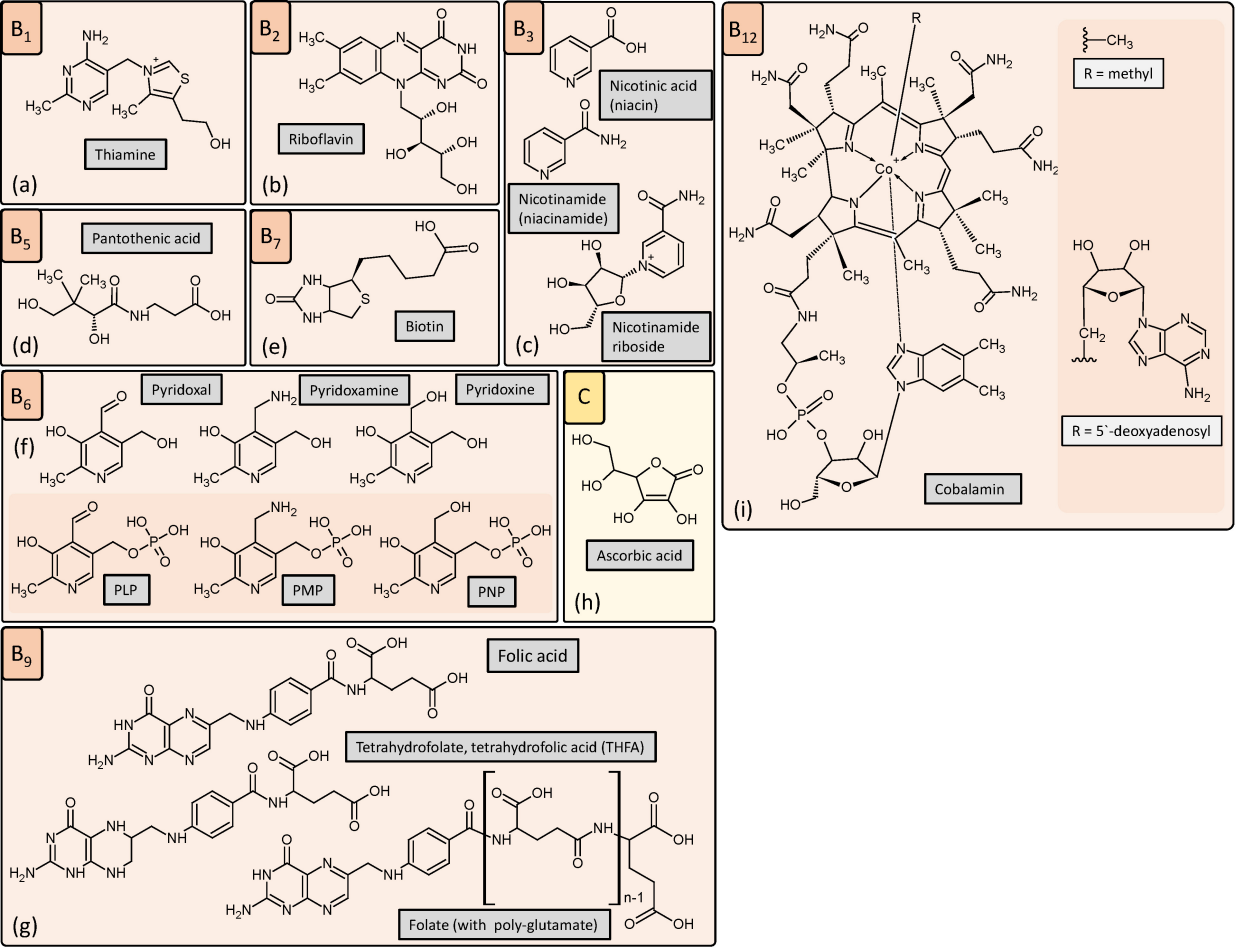
Chemical structures of the water-soluble vitamins B1, B2, B3, B5, B6, B7, B9, B12, C and vitamin groups. Created with ACD/ChemSketch (Freeware) 2021.1.1.

Chandra highlighted that nutrition, or more precisely a well-balanced diet containing antioxidant-, trace element- and vitamin-rich foods can counteract immunologic deterioration, e.g., by protecting immune cells against reactive oxygen species (ROS) (15). In turn, the lack of essential micronutrients reinforces the already enhanced susceptibility to common infections with an increase in symptom severity (16, 17). Vitamin C has first been identified as an effective treatment for scurvy. Ever since, the role of certain micronutrients, including zinc, iron, vitamins A, D, C and B12, among others, in synergistically and individually ensuring the proper functioning of our complex immune network has become increasingly evident. The need for their adequate dietary intake was emphasized by two review articles, since certain population groups such as the elderly are at higher risk of being deficient, which consequently results in impaired immunity and higher infection rates. The authors of these reviews also conclude that micronutrient deficiencies are a global public health problem (18, 19). In general, vitamins are categorized based on their solubility or storage properties (contrary to fat-soluble vitamins including vitamins A, D, E and K - water soluble vitamins are only stored to a limited extent and excreted via the urine) and are being produced by yeasts, plants and most commonly commensal intestinal bacteria. It has to be noted that humans in particular are not able to synthesize the majority of them and therefore rely on dietary vitamin intake (20, 21). Our recently published literature review, examining the influence of fat-soluble vitamins on immune parameters in the elderly, emphasized important aspects of how these particular micronutrients interact within the scope of our multifaceted immune system (22). Despite the established relevance of micronutrients in the human diet, specific immunomodulatory properties of water-soluble vitamins in the geriatric population remain insufficiently characterized. Due to high metabolic turnover, interindividual dietary intake and malabsorption along with limited storage capacity, the elderly represent a vulnerable cohort concerning subclinical deficiencies that might contribute to progressing immunosenescence. **Figure 1** illustrates the chemical structures of said vitamins, responsible for their general but also immunological properties, which will be discussed thoroughly in the following chapters. Therefore, following our recent literature review focusing on fat-soluble vitamins (22), this narrative review addresses state of the art scientific knowledge about water-soluble vitamins (B1-B12, C) regarding their potential to influence immune system parameters of older people upon depletion and repletion with the focus on maintaining immune resilience. In particular, this article aims to answer the questions how vitamin C and B-complex vitamins mechanistically influence innate and adaptive immune cells in the context of inflammation, to what extent targeted dietary supplementation could reverse age- or deficiency-related immunological impairments, and what specific clinical benefits this might offer in terms of infectious diseases for people aged 50 and older. By combining the insights of pre-clinical *in vitro* and animal-based studies with the scarce clinical trials, we provide a framework for understanding the complex interactions regarding the elderly, water-soluble vitamins, immunosenescence and inflammaging. To improve navigability for the reader we have created a table including relevant studies concerning immunologic aspects of the different water-soluble vitamins, structured by study type (**Supplementary Table 1**).

## 2 Methods

We conducted a comprehensive literature search across PubMed/MEDLINE, Scopus and Google Scholar to evaluate the immunomodulatory potential of water-soluble vitamins including B-complex vitamins (B1, B2, B3, B5, B6, B7, B9 and B12) and vitamin C, as well as their active vitamers, where applicable, in the aging population. Search strategies included the use of Boolean operators to categorize search results by thematic blocks focusing on the target population (search terms: elderly, seniors, individuals ages 50+, older people), immunologic parameters (search terms: immunosenescence, inflammaging, immune system, immune cells) and clinical outcomes (infection susceptibility, immunity). Inclusion criteria were defined by their relevance to age-related immunological status, deficiency states, or specific supplementation regimens. The scope encompassed randomized controlled trials and observational studies, complemented by animal models and *in vitro* research focusing on geriatric cohorts. Studies focusing on pediatric or healthy young adult populations, case reports and research not involving immunological markers as primary or secondary endpoint were excluded. To make the individual chapters on vitamins as accessible as possible, each chapter has been organized according to the following key areas: biochemical role and metabolism, prevalence of deficiency in the elderly, evidence for immunomodulatory effects, clinical implications and recommendations and summary, where applicable.

## 3 Thiamine (vitamin B1): role in immune function and aging

The first water-soluble vitamin we discuss is vitamin B1, also known as thiamine (**Figure 1A**), which is found in fairly high concentrations in, e.g., the husk and germ of cereal grains, beans, nuts, brown rice, pork loin or beef (23, 24). In accordance with its biochemical role and metabolism, functions in the body comprise the breakdown of carbohydrates along with potentially reducing the risk for age-related disorders such as metabolic syndrome or eye diseases (25–30). Thiamine pyrophosphate (TPP) represents the primary active coenzyme involved in the tricarboxylic acid (TCA) cycle, thereby playing an important role in energy metabolism regarding, for example, immune cell activation. Further phosphorylated biologically active derivatives of thiamine comprise thiamine triphosphate and adenosine thiamine triphosphate (20, 31–33). In this context, recent evidence proposes that homeostasis between glycolysis and TCA cycle is involved in the functional control of immune cells, the so-called “immunometabolism”; an example being naïve B cells which rely on the TCA cycle for energy production (32, 34). Further, thiamine impacts various immune cell populations which includes the thymic differentiation of T lymphocytes (34, 35) among other ramifications concerning macrophages, dendritic cells, neutrophils and thrombocytes (24).

**Figure 2:**
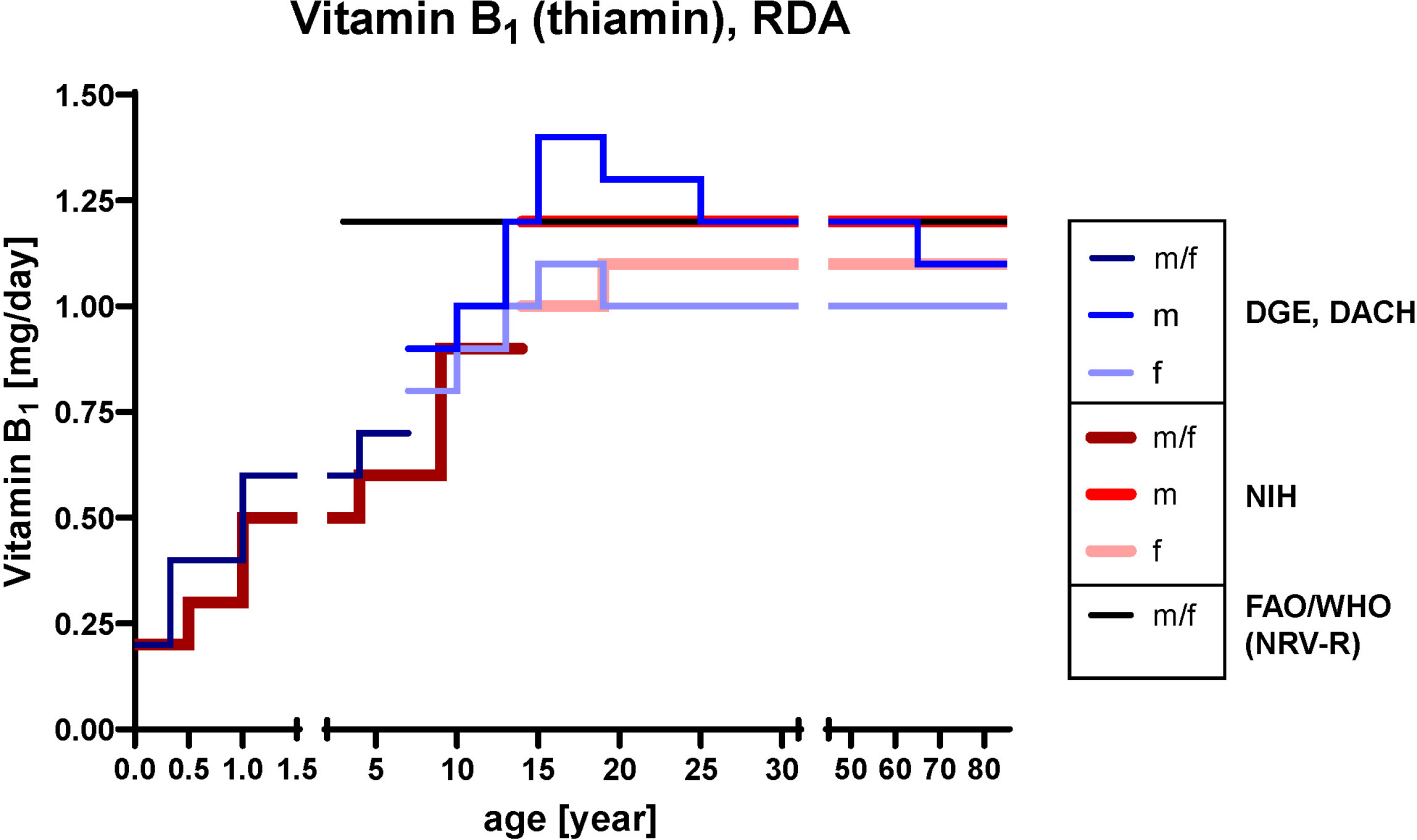
RDA reference values of vitamin B1. References according to D-A-CH [German Nutrition Society (DGE); Austrian Nutrition Society (ÖGE); Swiss Nutrition Society (SGE). Referenzwerte für die Nährstoffzufuhr [Dietary Reference Values], 2nd edition, 5th updated issue; German Nutrition Society: Bonn, Germany, 2019; ISBN 9783887492618]. DGE. Thiamin (Vitamin B1) (2025). Available at: https://www.dge.de/wissenschaft/referenzwerte/thiamin/ (Accessed May 26, 2025). (37)], NIH [Institute of Medicine. Dietary Reference Intakes for Thiamin, Riboflavin, Niacin, Vitamin B6, Folate, Vitamin B12, Pantothenic Acid, Biotin, and Choline. Washington: National Academies Press (2000) (38)] and the NRVs-R of FAO/WHO [Lewis J. Codex nutient reference values: Especially for vitamins, minerals and protein, p. 36. Rome. FAO and WHO (2019) (39)]. D-A-CH, Deutschland, Austria, Confoederatio Helvetica (eng. GSA, Germany, Switzerland, Austria); DGE, Deutsche Gesellschaft für Ernährung; FAO, Food and Agriculture Organization; NIH, National Institutes of Health; NRV-R, Nutrient Reference Value-Requirement; ÖGE, Österreichische Gesellschaft für Ernährung RDA, Recommended Dietary Allowance; SGE, Schweizerische Gesellschaft für Ernährung WHO, World Health Organization.

Even though an adequate thiamine status is supposed to be sustained by a well-balanced diet, different studies point out that older people tend to be poorly supplied with the vitamin, as it has been described in previous studies that 46% of the participating elderly (23) and 13–43% of community home residents had low storage or showed signs of deficiency (36). In close proximity, **Figure 2** and **Table 1** illustrate the RDA for vitamin B1 according to the different health institutions with recommendation for the elderly as well, whereas **Supplementary Figure 1** shows the percentage of people not having an adequate supply (20%–40% of people aged 65 and older below D-A-CH reference levels) based on data of the German National Nutrition Survey II (2005–2007) (46), which corresponds to the data presented above. Data summarized in Table 1 was collected using the official homepages and statements from the issuing organizations (38–43, 47).

**Table 1:** Intake recommendations for water-soluble vitamins (people aged 50+).

	Issuing Organization					
	DGE	D-A-CH	NVS II (D-A-CH)	NRVs	DRIs (RDA)	DRIs (UL)
References	(40)	(41)	(42)	(39)	(38, 43)	(38, 43)
Thiamine: Vitamin B1	Female: 51-64 y/o (1.0 mg) 65+ y/o (1.0 mg) Male: 51-64 y/o (1.2 mg) 65+ y/o (1.1 mg)	Female: 51-64 y/o (1.0 mg) 65+ y/o (1.0 mg) Male: 51-64 y/o (1.2 mg) 65+ y/o (1.1 mg)	Female: 51-64 y/o (1.0 mg) 65-80 y/o (1.0 mg) Male: 51-64 y/o (1.1 mg) 65-80 y/o (1.0 mg)	General population > 36 months y/o (1.2 mg)	Female: 51-70 y/o (1.1 mg) 70+ y/o (1.1 mg) Male: 51-70 y/o (1.2 mg) 70+ y/o (1.2 mg)	Female: 51-70 y/o (n/a) 70+ y/o (n/a) Male: 51-70 y/o (n/a) 70+ y/o (n/a)
Riboflavin: Vitamin B2	Female: 51-64 y/o (1.0 mg) 65+ y/o (1.0 mg) Male: 51-64 y/o (1.3 mg) 65+ y/o (1.3 mg)	Female: 51-64 y/o (1.0 mg) 65+ y/o (1.0 mg) Male: 51-64 y/o (1.3 mg) 65+ y/o (1.3 mg)	Female: 51-64 y/o (1.2 mg) 65-80 y/o (1.2 mg) Male: 51-64 y/o (1.3 mg) 65-80 y/o (1.2 mg)	General population > 36 months y/o (1.2 mg)	Female: 51-70 y/o (1.1 mg) 70+ y/o (1.1 mg) Male: 51-70 y/o (1.3 mg) 70+ y/o (1.3 mg)	Female: 51-70 y/o (n/a) 70+ y/o (n/a) Male: 51-70 y/o (n/a) 70+ y/o (n/a)
Niacin: Vitamin B3	Female: 51-64 y/o (11 mg NE) * 65+ y/o (11 mg NE) * Male: 51-64 y/o (15 mg NE) * 65+ y/o (14 mg NE) *	Female: 51-64 y/o (11 mg NE) * 65+ y/o (11 mg NE) * Male: 51-64 y/o (15 mg NE) * 65+ y/o (14 mg NE) *	Female: 51-64 y/o (13 mg) 65-80 y/o (13 mg) Male: 51-64 y/o (15 mg) 65-80 y/o (13 mg)	General population > 36 months y/o (15 mg NE) *	Female: 51-70 y/o (14 mg NE) * 70+ y/o (14 mg NE) * Male: 51-70 y/o (16 mg NE) * 70+ y/o (16 mg NE) *	Female: 51-70 y/o (35 mg NE) * 70+ y/o (35 mg NE) * Male: 51-70 y/o (35 mg NE) * 70+ y/o (35 mg NE) *
Pantothenic acid: Vitamin B5	Female: 51-64 y/o (5 mg) 65+ y/o (5 mg) Male: 51-64 y/o (5 mg) 65+ y/o (5 mg)	Female: 51-64 y/o (6 mg) 65+ y/o (6 mg) Male: 51-64 y/o (6 mg) 65+ y/o (6 mg)	Female: 51-64 y/o (n/a) 65-80 y/o (n/a) Male: 51-64 y/o (n/a) 65-80 y/o (n/a)	General population > 36 months y/o (5 mg)	Female: 51-70 y/o (5 mg) 70+ y/o (5 mg) Male: 51-70 y/o (5 mg) 70+ y/o (5 mg)	Female: 51-70 y/o (n/a) 70+ y/o (n/a) Male: 51-70 y/o (n/a) 70+ y/o (n/a)
Pyridoxine: Vitamin B6	Female: 51-64 y/o (1.4 mg) 65+ y/o (1.4 mg) Male: 51-64 y/o (1.6 mg) 65+ y/o (1.6 mg)	Female: 51-64 y/o (1.2 mg) 65+ y/o (1.2 mg) Male: 51-64 y/o (1.5 mg) 65+ y/o (1.4 mg)	Female: 51-64 y/o (1.2 mg) 65-80 y/o (1.2 mg) Male: 51-64 y/o (1.5 mg) 65-80 y/o (1.4 mg)	General population > 36 months y/o (1.3 mg)	Female: 51-70 y/o (1.5 mg) 70+ y/o (1.5 mg) Male: 51-70 y/o (1.7 mg) 70+ y/o (1.7 mg)	Female: 51-70 y/o (100 mg) 70+ y/o (100 mg) Male: 51-70 y/o (100 mg) 70+ y/o (100 mg)
Biotin: Vitamin B7	Female: 51-64 y/o (40 µg) 65+ y/o (40 µg) Male: 51-64 y/o (40 µg) 65+ y/o (40 µg)	Female: 51-64 y/o (30-60 µg) 65+ y/o (30-60 µg) Male: 51-64 y/o (30-60 µg) 65+ y/o (30-60 µg)	Female: 51-64 y/o (n/a) 65-80 y/o (n/a) Male: 51-64 y/o (n/a) 65-80 y/o (n/a)	General population > 36 months y/o (30 µg)	Female: 51-70 y/o (30 µg) 70+ y/o (30 µg) Male: 51-70 y/o (30 µg) 70+ y/o (30 µg)	Female: 51-70 y/o (n/a) 70+ y/o (n/a) Male: 51-70 y/o (n/a) 70+ y/o (n/a)
Folate: Vitamin B9	Female: 51-64 y/o (300 µg DFE) ** 65+ y/o (300 µg DFE) ** Male: 51-64 y/o (300 µg DFE) ** 65+ y/o (300 µg DFE) **	Female: 51-64 y/o (300 µg DFE) ** 65+ y/o (300 µg DFE) ** Male: 51-64 y/o (300 µg DFE) ** 65+ y/o (300 µg DFE) **	Female: 51-64 y/o (400 µg DFE) ** 65-80 y/o (400 µg DFE) ** Male: 51-64 y/o (400 µg DFE) ** 65-80 y/o (400 µg DFE) **	General population > 36 months y/o (400 µg DFE) **	Female: 51-70 y/o (400 µg DFE) ** 70+ y/o (400 µg DFE) ** Male: 51-70 y/o (400 µg DFE) ** 70+ y/o (400 µg DFE) **	Female: 51-70 y/o (1000 µg) 70+ y/o (1000 µg) Male: 51-70 y/o (1000 µg) 70+ y/o (1000 µg)
Cobalamin: Vitamin B12	Female: 51-64 y/o (4 µg) 65+ y/o (4 µg) Male: 51-64 y/o (4 µg) 65+ y/o (4 µg)	Female: 51-64 y/o (3 µg) 65+ y/o (3 µg) Male: 51-64 y/o (3 µg) 65+ y/o (3 µg)	Female: 51-64 y/o (3 µg) 65-80 y/o (3 µg) Male: 51-64 y/o (3 µg) 65-80 y/o (3 µg)	General population > 36 months y/o (2.4 µg)	Female: 51-70 y/o (2.4 µg) 70+ y/o (2.4 µg) Male: 51-70 y/o (2.4 µg) 70+ y/o (2.4 µg)	Female: 51-70 y/o (n/a) 70+ y/o (n/a) Male: 51-70 y/o (n/a) 70+ y/o (n/a)
Ascorbate: Vitamin C	Female: 51-64 y/o (95 mg) 65+ y/o (95 mg) Male: 51-64 y/o (110 mg) 65+ y/o (110 mg)	Female: 51-64 y/o (95 mg) 65+ y/o (95 mg) Male: 51-64 y/o (110 mg) 65+ y/o (110 mg)	Female: 51-64 y/o (100 mg) 65-80 y/o (100 mg) Male: 51-64 y/o (100 mg) 65-80 y/o (100 mg)	General population > 36 months y/o (100 mg)	Female: 51-70 y/o (75 mg) 70+ y/o (75 mg) Male: 51-70 y/o (90 mg) 70+ y/o (90 mg)	Female: 51-70 y/o (2000 mg) 70+ y/o (2000 mg) Male: 51-70 y/o (2000 mg) 70+ y/o (2000 mg)

D-A-CH, Deutschland, Austria, Confoederatio Helvetica (eng. GSA, Germany, Switzerland, Austria); DFE, dietary folate equivalents; DGE, Deutsche Gesellschaft für Ernährung (eng. German Nutrition Society); DRI, Dietary Reference Intake; n/a, not available; NE, niacin equivalents; NRV, Nutrient Reference Values; NVS, Nationale Verzehrsstudie (eng. National Nutrition Survey); RDA, Recommended Dietary Allowance; UL, Tolerable Upper Intake Level; y/o, years old.

*1 mg NE = 1 mg niacin or 60 mg tryptophan (39, 44).

**1 µg DFE = 1 µg folate coming from food; 0.6 µg folic acid added to food or as supplement consumed with food; 0.5 µg synthetic folic acid (folic acid added to food or as supplement taken on an empty stomach) (39, 45).

https://www.ncbi.nlm.nih.gov/books/NBK56068/table/summarytables.t2/?report=objectonly (last access: 06.11.2025).

https://www.ncbi.nlm.nih.gov/books/NBK56068/table/summarytables.t7/?report=objectonly (last access: 06.11.2025).

Clinical vitamin B1 deficiency can manifest as beriberi or Wernicke-Korsakoff syndrome, both disorders affecting the nervous- and cardiovascular system. These disorders are often accompanied by chronic neuroinflammation and have been associated with multiple neurodegenerative diseases like Alzheimer’s or Parkinson’s disease. *In vitro* as well as *in vivo* research provides further insight regarding the underlying immunologic consequences of thiamine deficiency comprising a decreased number of naïve B cells in Peyer’s patches, increased T cell infiltration or the overshooting expression of inflammation-promoting cytokines such interleukin-6 (IL-6) and tumor necrosis factor alpha (TNF-α). Moreover, preclinical studies report a decrease in the phagocytic activity of peripheral blood leukocytes as well as compromised antibody production and reduced bactericidal activity in the serum under deficient conditions (33, 34, 48–52).

Concerning immunity enhancing properties, thiamine has been shown to counteract pro-inflammatory responses. In this regard, Bozic et al. found out that benfotiamine, a synthetic vitamin B1 precursor, was able to significantly decrease the inflammatory environment in lipopolysaccharides (LPS) stimulated microglial cells, e.g., by inhibiting the expression of inducible nitric oxide synthase (iNOS) and nitric oxide (NO) just to name a few (53). Similar anti-inflammatory and pathogen-clearing effects could be observed by (54). Benfotiamine inhibited the production of prostaglandin E2 (PGE2), nuclear factor kappa-light-chain-enhancer of activated B cells (NF-kB), cyclooxygenase-2 (COX-2) and cell death among other parameters in LPS stimulated murine RAW264.7 macrophages (54). Olkowski et al. (55) on the other hand described that thiamine enhances neutrophilic activity against *Candida albicans*. The vitamin also participates in immune cell localization by facilitating the release of intracellular adhesion molecules (ICAMs) among protecting the cells against oxidative damage (56). Studies investigating the immunologic impact found evidence of low vitamin B1 levels being related to compromised lymphocyte count and function as well as phagocytic activity, whereas benfotiamine supplementation showed potential activity against advanced glycation end products (AGE) characteristics, perhaps by restoring immune homeostasis in patients with diabetes (57).

In summary, thiamine is an important but broadly neglected B-vitamin concerning intervention studies, RCTs in particular, especially regarding its impact on “immunometabolism” in the elderly, whereas it is commonly known that its active derivative TPP plays an important role in the energy metabolism and functionality of different immune cell populations. Nonetheless, many elderly people face problems with adequate supply and states of deficiency, leading to inflammatory responses and various negative immunologic consequences. To date, research mainly relies on cell culture and animal-based models or the use of a multi-nutrient supplement, which highlights the need for future studies on vitamin B1’s independent impact on immunosenescence or vaccination efficacy respectively, focusing on human trials and omics-technologies, in order to identify adequate requirements for maintaining immune resilience in the aging population.

## 4 Riboflavin (vitamin B2): role in immune function and aging

Riboflavin or vitamin B2 is the second vitamin that is being discussed. Its chemical structure is illustrated in **Figure 1B** and general food sources comprise dark-green vegetables, fruits, eggs, dairy products or meat, whereas main functions in the body include the maintenance of erythrocyte synthesis, metabolic involvement, skin health and its biochemical function as an antioxidant and as a cofactor for different enzymes associated with energy metabolism and the TCA (riboflavin acts as a precursor for flavin mononucleotide (FMN), which is converted into flavin adenine dinucleotide (FAD)), along with cardiovascular and neurological health (25, 34, 58–60).

**Figure 3:**
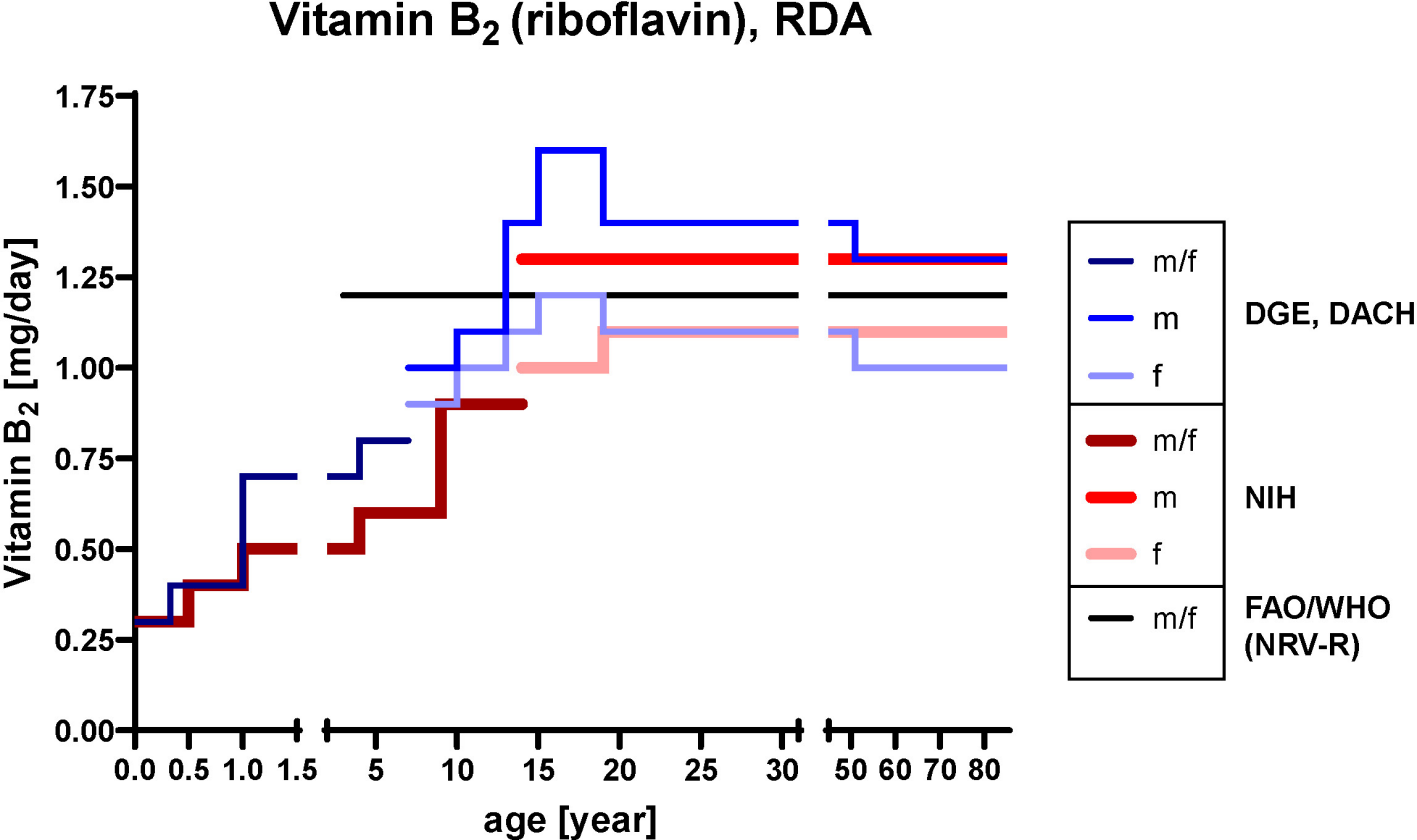
RDA reference values of vitamin B2. References according to D-A-CH [German Nutrition Society (DGE); Austrian Nutrition Society (ÖGE); Swiss Nutrition Society (SGE). Referenzwerte für die Nährstoffzufuhr [Dietary Reference Values], 2nd edition, 5th updated issue; German Nutrition Society: Bonn, Germany (2019); ISBN 9783887492618.], DGE [DGE. Riboflavin (Vitamin B2) (2025). Available at: https://www.dge.de/wissenschaft/referenzwerte/riboflavin/ (Accessed May 26, 2025). (63)], NIH [Institute of Medicine. Dietary Reference Intakes for Thiamin, Riboflavin, Niacin, Vitamin B6, Folate, Vitamin B12, Pantothenic Acid, Biotin, and Choline. Washington: National Academies Press (2000) (38)] and the NRVs-R of FAO/WHO [Lewis J. Codex nutient reference values: Especially for vitamins, minerals and protein, p. 36. Rome. FAO and WHO (2019) (39)]. D-A-CH, Deutschland, Austria, Confoederatio Helvetica (eng. GSA, Germany, Switzerland, Austria); DGE, Deutsche Gesellschaft für Ernährung (eng. German Nutrition Society); FAO, Food and Agriculture Organization; NIH, National Institutes of Health; NRV-R, Nutrient Reference Value-Requirement; ÖGE, Österreichische Gesellschaft für Ernährung RDA, Recommended Dietary Allowance; SGE, Schweizerische Gesellschaft für Ernährung WHO, World Health Organization.

Inadequate supply via diet or supplements as well as physiological stress potentially results in riboflavin deficiency which manifests, e.g., through migraines, stomatitis, depression or cognitive disorders (25). Concomitantly, a poor vitamin B2 status tends to be of importance regarding, among other risk groups like alcoholics, the elderly in particular (compare Powers et al. (61): 41% of free-living elderly people were reported as being B2-deficient (61, 62)). Respectively, **Figure 3** as well as **Table 1** illustrate the RDA for vitamin B2 according to the different health institutions with recommendation for the elderly as well, whereas **Supplementary Figure 2** shows the percentage of people not having an adequate supply (20%–30% of people aged 65 and older below D-A-CH reference levels) based on data of the German National Nutrition Survey II (2005–2007) (64).

Immunologic consequences originating from low riboflavin levels encompass most prominently inflammation, which was shown by Mazur-Bialy et al. (62) and Mazur-Bialy and Pocheć (65) who examined the role of vitamin B2 on RAW264.7 macrophages, describing that deficiency-induced pro inflammatory effects such as the release of TNF-α, IL-1, iNOS and monocyte chemoattractant protein-1 (MCP-1) among others could be reversed upon riboflavin supplementation. Similar results were demonstrated by Dey and Bishayi (66). Riboflavin administration was able to counteract the release of pro-inflammatory cytokines such as IL-6, interferon gamma (IFN-γ) or NO while simultaneously enhancing phagocytosis in *Staphylococcus aureus* infected macrophages. Interestingly, combining antigenic gut-bacterial-produced vitamin B2 metabolites with the major histocompatibility complex class I-related gene protein (MR1) leads to the activation of mucosal-associated invariant T (MAIT) cells which play an important role in intestinal inflammation, mucosal defense and gut immune homeostasis (67–69). Further immunologic impacts of riboflavin include anti-inflammatory effects such as the reduction of IL-1β, IL-6 and IFN γ (61) and reverse outcomes upon vitamin B2 deprivation including the decrease of the proliferation rate as well as inhibition of respiratory burst of mouse monocytes and macrophages. Mazur-Bialy and Pocheć revealed adipocyte death and increased ROS-, NF-kB-, TNF-α - and IL-6-levels in mycoplasma-free mouse preadipocytes with riboflavin deficiency (3.1 nM) in contrast to control conditions (10.4 nM) (70, 71). Mikkelsen and Apostolopoulos (34) cited a clinical study administering riboflavin that found evidence of enhanced neutrophil-, monocyte- and macrophage number and activity leading to an increased resilience against *E. coli* infections.

In summary, riboflavin significantly contributes to various aspects concerning immunocompetence of the elderly, impacting mitochondrial energy production, the regulation of innate immune responses like activating MAIT cells or antioxidant defense and the inhibition of pro-inflammatory signaling, just to name a few. Regarding the discrepancy between vitamin B2’s relevance for the immune system and prevalent inadequate supply or deficiency in older populations, future studies need to address the question whether current intake recommendations meet the physiological needs of the elderly for maintaining immune function. To the best of our knowledge, RCTs and human *in vivo* studies investigating the immunomodulatory potential of riboflavin concerning older individuals are scarce, as most published studies include cell-culture based *in vitro* approaches, focusing on inflammatory effects in the context of vitamin B2 deficiency and concomitantly the impact of supplementation. Therefore, geriatric-specific clinical trials need to be conducted to establish targeted supplementation protocols and identify riboflavin’s potential in reducing infectious morbidity and neuroinflammation while restoring immune homeostasis and magnifying pathogenic resilience in the elderly.

## 5 Niacin (vitamin B3): role in immune function and aging

Vitamin B3, also known as nicotinic acid or niacin (**Figure 1C**), needs to be provided via diet (fish, meat, mushrooms or beans), although the less efficient endogenous synthesis (1 mg niacin equivalents (NE) = 1 mg niacin or 60 mg tryptophan; **Table 1**) happens due to consuming the essential proteinogenic amino acid tryptophan, and participates in a variety of biochemical processes including energy-, fatty acid as well as cholesterol production, skin health or DNA protection (20, 72, 73).

**Figure 4:**
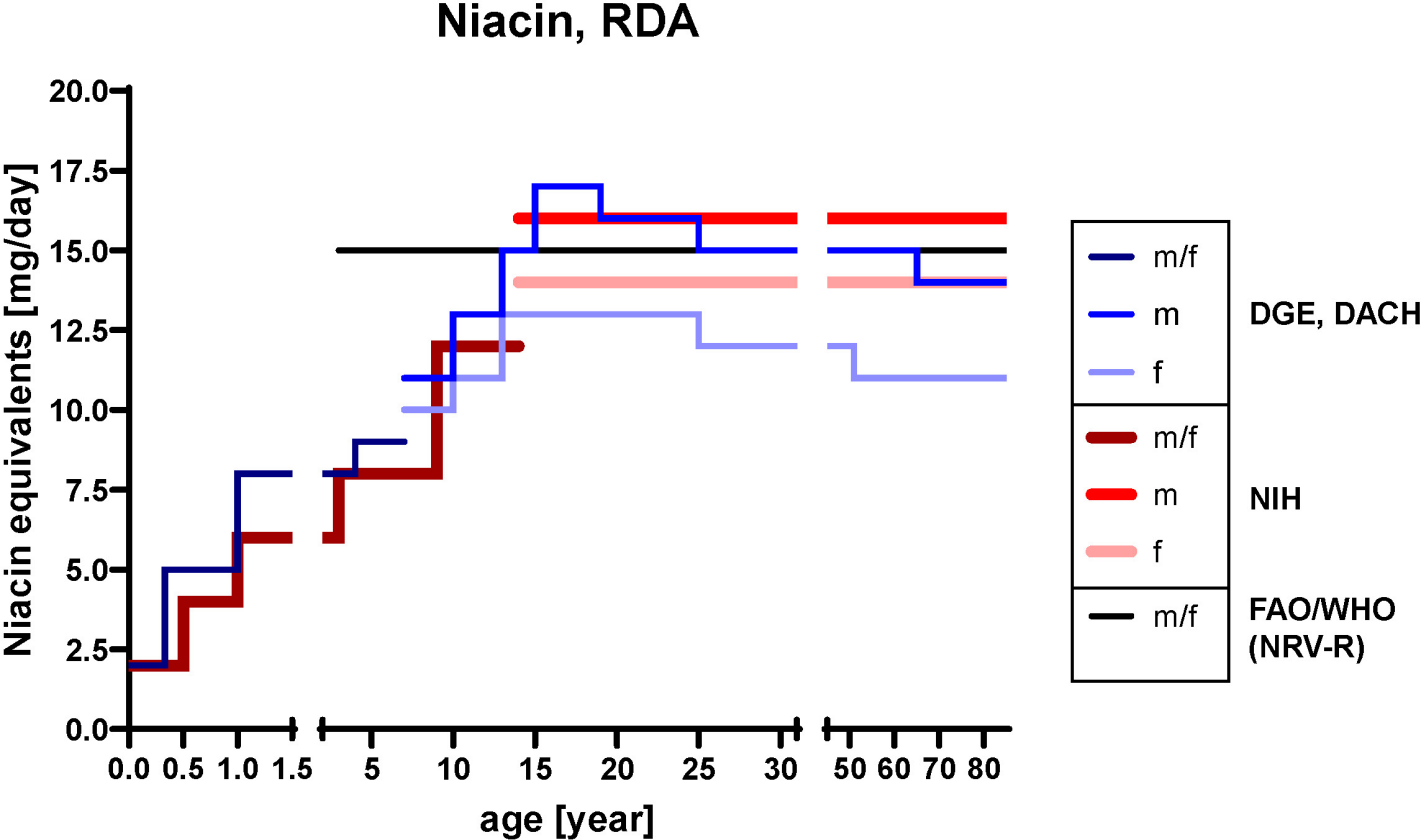
RDA reference values of vitamin B3 (niacin). References according to D-A-CH [German Nutrition Society (DGE); Austrian Nutrition Society (ÖGE); Swiss Nutrition Society (SGE). Referenzwerte für die Nährstoffzufuhr [Dietary Reference Values], 2nd edition, 5th updated issue; German Nutrition Society: Bonn, Germany, 2019; ISBN 9783887492618.], DGE [DGE. Niacin (2025). Available at: https://www.dge.de/wissenschaft/referenzwerte/niacin/ (Accessed May 26, 2025). (44)], NIH [Institute of Medicine. Dietary Reference Intakes for Thiamin, Riboflavin, Niacin, Vitamin B6, Folate, Vitamin B12, Pantothenic Acid, Biotin, and Choline. Washington: National Academies Press (2000) (38)] and the NRVs-R of FAO/WHO [Lewis J. Codex nutient reference values: Especially for vitamins, minerals and protein, p. 36. Rome. FAO and WHO (2019) (39)]. D-A-CH, Deutschland, Austria, Confoederatio Helvetica (eng. GSA, Germany, Switzerland, Austria); DGE, Deutsche Gesellschaft für Ernährung (eng. German Nutrition Society); FAO, Food and Agriculture Organization; NIH, National Institutes of Health; NRV-R, Nutrient Reference Value-Requirement; ÖGE, Österreichische Gesellschaft für Ernährung RDA, Recommended Dietary Allowance; SGE, Schweizerische Gesellschaft für Ernährung WHO, World Health Organization.

Accordingly, deficiency symptoms, although not very common, comprise dementia, skin disorders, depression, somatic complications or pellagra, which appear to be reversible upon niacin supplementation due to its inflammation-, and lipid-lowering or cardioprotective effects (72, 74, 75). Respectively, **Figure 4** as well as **Table 1** illustrate the RDA for vitamin B3 according to the different health institutions with recommendation for the elderly as well, whereas **Supplementary Figure 3** shows the percentage of people not having an adequate supply (less than 10% of people aged 65 and older below D-A-CH reference levels) based on data of the German National Nutrition Survey II (2005–2007) (76), which corresponds to the data of deficiency occurring only in rare occasions.

Regarding its immunomodulatory potential, vitamin B3 showed anti-inflammatory properties regarding the brain (neuroinflammatory diseases), skin and gastrointestinal tract and modulated certain immunologic parameters such as the differentiation of monocytes into M2 anti-inflammatory macrophages. Concomitantly, the expression of the nicotinic acid receptor NIACR1 (GPR109A), a G-protein coupled receptor, on the surface of adipocytes, but also immune cells such as macrophages, monocytes, neutrophils and dendritic cells, seems to play an important role in neuroprotective mechanisms along with the decrease of monocyte chemotaxis and pro-inflammatory cytokines like IL-6, TNF-α, MCP-1 and NF-κB (77–81). Moreover, receptor activation results in the differentiation of regulatory T cells (Tregs) (82).

Regarding the clinical relevance, a number of *in vivo* as well as *in vitro* studies that are reviewed by Mitra et al. supports immunomodulation by vitamin B3 by targeting inflammatory and oxidative stress related processes (71). Different studies suggest that vitamin B3 enhances the innate immune responses up to 1000-fold, thereby improving the body’s response to *S. aureus* infections together with the already mentioned anti-inflammatory effects concerning vascular or experimental atheromatous inflammation (80, 83, 84). Niacin was able to abate neutrophil infiltration in persons with lung injury caused by ventilators and also impaired replication of certain viruses including hepatitis B and human immunodeficiency virus among others (85). According to Rawji et al., monocyte-derived macrophages and microglia are important for remyelination in the context of, e.g., multiple sclerosis, but during the process of aging they exhibit delayed responses resulting in lower effectiveness. Supplementation with niacin seems to stimulate scavenger receptor CD36 expression, thereby enhancing myelin phagocytosis and ultimately remyelination (86).

In summary, clinical niacin deficiency in the elderly occurs seldomly which resembles an overall adequate supply status. Examples for the vitamin B3’s immune effects are the reduction of inflammatory processes related to “inflammaging”, the positive impact on differentiation and activity of certain immune cell populations and an enhanced innate host defense including increased pathogen clearance. There are only few studies investigating the effect of niacin on immune parameters in the elderly. Most studies focus on vitamin B3’s anti-inflammatory properties on a cellular- or animal-based level, rather than standardized clinical trials pinpointing the potential of niacin in reversing immunosenescence in older adults or providing a biochemical strategy to enhance geriatric immunocompetence via an eligible supplementation regimen supposedly above current dietary intake recommendations. Therefore, future investigations must address these uncertainties in order to fill the knowledge gaps regarding the aging immune system and vitamin B3.

## 6 Pantothenic acid (vitamin B5): role in immune function and aging

Abundant dietary sources of pantothenic acid (**Figure 1D**) include legumes, eggs, avocado, yeast, cereal grains, milk, vegetables and meat among others (26, 71), whereas most prevalent biochemical functions comprise energy metabolism by contributing to coenzyme A (CoA) synthesis (cofactor in TCA cycle and fatty acid metabolism), erythropoiesis and maintenance of nervous system (26, 56, 87, 88). The increase of CoA activity contributes to immunological changes such as enhanced acute-phase proteins, corresponding to altered defense mechanisms (89, 90).

**Figure 5:**
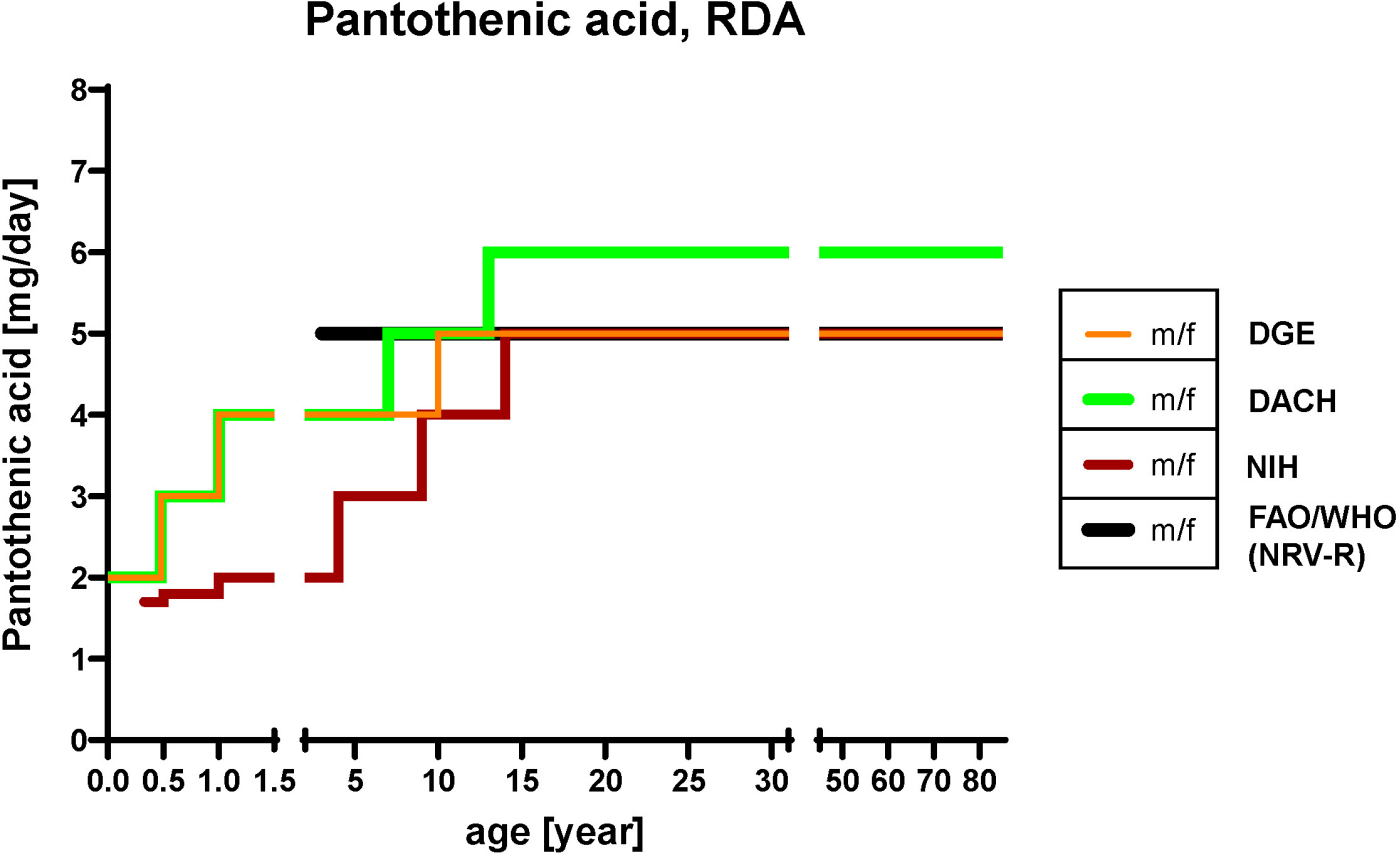
RDA reference values of vitamin B5 (pantothenic acid). References according to D-A-CH [German Nutrition Society (DGE); Austrian Nutrition Society (ÖGE); Swiss Nutrition Society (SGE). Referenzwerte für die Nährstoffzufuhr [Dietary Reference Values], 2nd edition, 5th updated issue; German Nutrition Society: Bonn, Germany, 2019; ISBN 9783887492618. (Deutsche Gesellschaft für Ernährung et al., 2019)], DGE [DGE. Pantothensäure (2025). Available at: https://www.dge.de/wissenschaft/referenzwerte/pantothensaeure/ (Accessed May 26, 2025). (91)], NIH [Institute of Medicine. Dietary Reference Intakes for Thiamin, Riboflavin, Niacin, Vitamin B6, Folate, Vitamin B12, Pantothenic Acid, Biotin, and Choline. Washington: National Academies Press (2000) (38)] and the NRVs-R of FAO/WHO [Lewis J. Codex nutient reference values: Especially for vitamins, minerals and protein. Rome: FAO and WHO (2019) (39)]. D-A-CH, Deutschland, Austria, Confoederatio Helvetica (eng. GSA, Germany, Switzerland, Austria); DGE, Deutsche Gesellschaft für Ernährung (eng. German Nutrition Society); FAO, Food and Agriculture Organization; NIH, National Institutes of Health; NRV-R, Nutrient Reference Value-Requirement; ÖGE, Österreichische Gesellschaft für Ernährung RDA, Recommended Dietary Allowance; SGE, Schweizerische Gesellschaft für Ernährung WHO, World Health Organization.

**Figure 5** as well as **Table 1** illustrate the RDA for vitamin B5 according to the different health institutions with recommendations for the elderly as well. Although not very common, deficiency symptoms supposedly involve higher susceptibility to respiratory tract infections, depression, inflammation, dermatitis and potentially neurodegeneration and Alzheimer’s disease (26, 92–94). Interestingly, vitamin B5 and vitamin D_3_ deficiency seem to be linked due to altered intestinal bacterial B-vitamin (including pantothenate) production, potentially caused by low calcitriol levels, which, in turn, is associated with numerous diseases, such as rheumatoid arthritis or atherosclerosis (34, 92).

Apart from that, pantothenic acid has been shown to exhibit immune-modulating effects, since the treatment of *Mycobacterium tuberculosis* (strain H37Rv) infected rodents led to an increase in macrophage-induced phagocytosis (decreased number of colony-forming units in lungs) as well as an elevated secretion of IFN-γ and IL-17 by CD4+ T cells, whereas the overall percentages of CD4+ and CD8+ T cells and polymorphic nuclear neutrophils could notxbe altered (95). In contrast, another study reported that the water-soluble vitamin seems to enhance CD8+ cytotoxic T cell differentiation into IL-22 producing Tc22, antitumor effector cells, which might play an essential role concerning anticancer immunosurveillance (96).

Accordingly, the vitamin B5 derivative dexpanthenol was able to diminish inflammation as well as oxidative stress partially by enhancing antioxidant enzymes such as glutathione or superoxide dismutase (97–99). Lastly, pantothenate contributes to Th1 and Th17 cell differentiation, macrophage maturation, epithelial TNF-α and IL-6 secretion and maintenance of gut mucosal barrier in mice with inflammatory bowel disease (100–103).

Clinically, Jung et al. found proof of pantothenic acid displaying anti-inflammatory features including a correlation between higher pantothenic acid intake and lower serum c-reactive protein (CRP) levels in older individuals (104).

In summary, pantothenic acid is characterized by an overall sufficient supply status and plays an important role in the immunometabolism with various effects on immune cell activation and differentiation as well as reinforcing anti-inflammatory and antioxidative defense systems. However, due to its broad availability through diet and rarely occurring deficiency, pantothenic acid has not been studied extensively in this context (56) while mechanistic insights mainly rely on *in vitro* or animal-based investigations. This might explain why only few studies exist concerning immunologic consequences of vitamin B5 intake-, deficiency or supplementation with particular focus on the elderly. Up to date, there is no standardized average intake requirement for geriatric populations regarding the optimization of their immune resilience, which might be of great importance for institutionalized elderly at risk of malnutrition, since B5 deficiency correlates with an increased susceptibility toward respiratory infections and neurodegenerative pathologies. Hence, there is a great demand for RCTs identifying pantothenic acid’s contribution to a properly functioning immune system in polymedicated older adults regarding nutrient-drug interactions as well as in the healthy elderly.

## 7 Pyridoxine (vitamin B6): role in immune function and aging

The term vitamin B6 (**Figure 1F**) encompasses the following compounds: pyridoxine, pyridoxamine, pyridoxal, pyridoxal-5-phosphate (PLP; active form), 4-pyridoxic acid and pyridoxine hydrochloride (34, 105). Main dietary sources comprise animal as well as plant-based foods including tuna, beef, liver, salmon, rice, chickpeas, starchy vegetables, non-citrus fruits, soy-based meat substitutes or fortified cereals (38, 106). Adequate consumption is indispensable as the vitamin is involved in many biochemical pathways, such as hemoglobin-synthesis, the production of various neurotransmitters including serotonin, dopamine, melatonin, norepinephrine, endorphin or gamma-aminobutyric acid (GABA) as well as lymphocyte differentiation, proliferation and maturation, in addition to its anti-inflammatory and antibacterial properties (18, 19, 34, 107).

**Figure 6:**
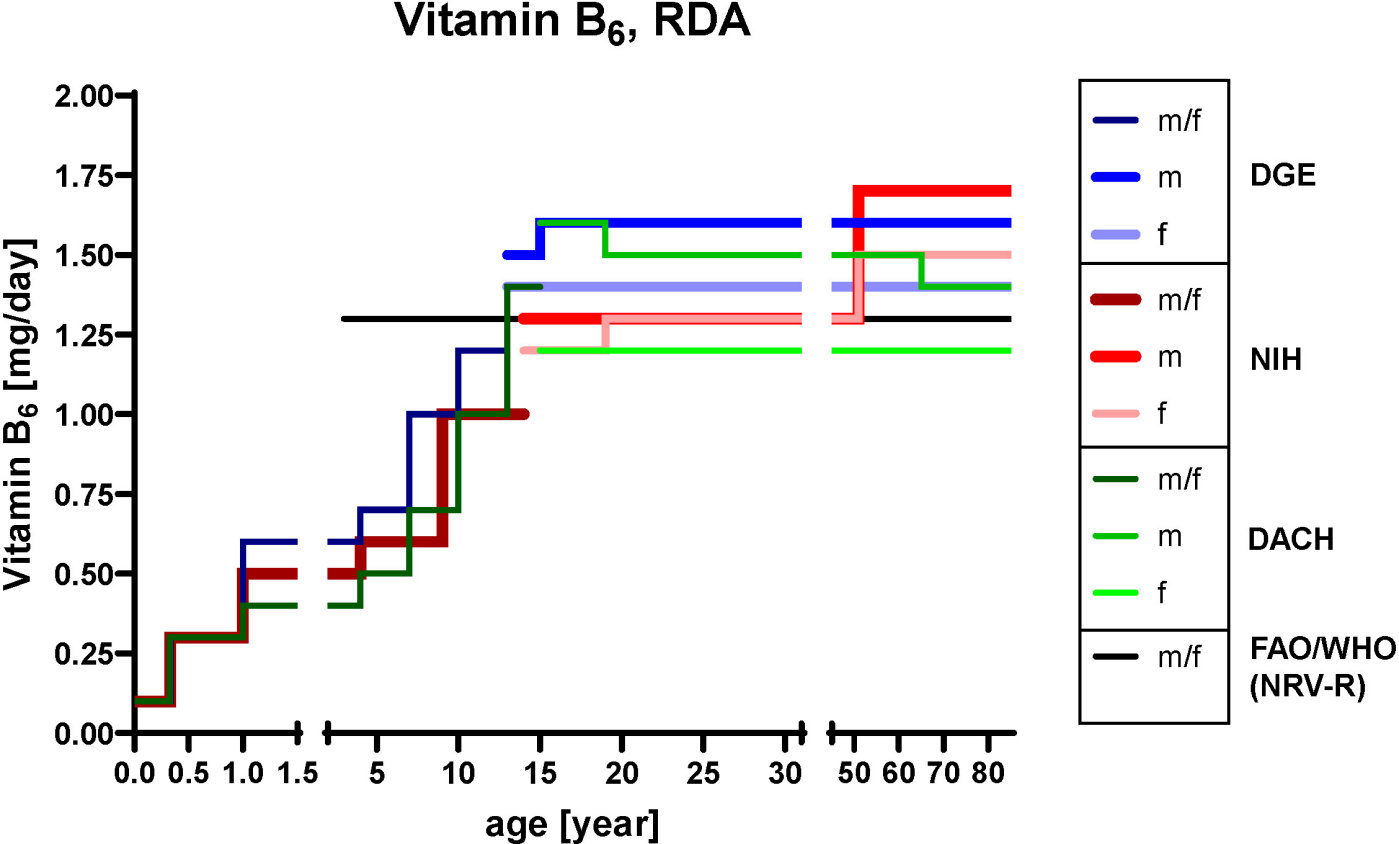
RDA reference values of vitamin B6. References according to D-A-CH [German Nutrition Society (DGE); Austrian Nutrition Society (ÖGE); Swiss Nutrition Society (SGE). Referenzwerte für die Nährstoffzufuhr [Dietary Reference Values], 2nd edition, 5th updated issue; German Nutrition Society: Bonn, Germany, 2019; ISBN 9783887492618.], DGE. Vitamin B6 (2025). Available at: https://www.dge.de/wissenschaft/referenzwerte/vitamin-b6/ (Accessed May 26, 2025). NIH [Institute of Medicine. Dietary Reference Intakes for Thiamin, Riboflavin, Niacin, Vitamin B6, Folate, Vitamin B12, Pantothenic Acid, Biotin, and Choline. Washington: National Academies Press (2000)] (38) and the NRVs-R of FAO/WHO [Lewis J. Codex nutient reference values: Especially for vitamins, minerals and protein, p. 36. Rome. FAO and WHO (2019) (39)]. D-A-CH, Deutschland, Austria, Confoederatio Helvetica (eng. GSA, Germany, Switzerland, Austria); DGE, Deutsche Gesellschaft für Ernährung (eng. German Nutrition Society); FAO, Food and Agriculture Organization; NIH, National Institutes of Health; NRV-R, Nutrient Reference Value-Requirement; ÖGE, Österreichische Gesellschaft für Ernährung RDA, Recommended Dietary Allowance; SGE, Schweizerische Gesellschaft für Ernährung WHO, World Health Organization.

Respectively, **Figure 6** as well as **Table 1** (38–43, 108) illustrate the RDA for vitamin B6 according to the different health institutions with recommendation for the elderly as well, whereas **Supplementary Figure 4** shows the percentage of people not having an adequate supply (around 15% of people aged 65 and older below D-A-CH reference levels) based on data of the German National Nutrition Survey II (2005–2007) (109). Next to dermatitis, depression, confusion and anemia, compromised immunity depicts a noteworthy manifestation of vitamin B6 deficiency (106), especially concerning the elderly, as they represent one of the population groups featuring a suppressed immune system and an age-related decline in B6 status (compare Gay and Meydani (110)).

Along with that, low vitamin B6 status causes impaired antibody production and response of delayed-type hypersensitivity (DTH), IL-1β, IL-2 (-receptor), NK-cell activity, altered T cell responses as well as a shift from anti- to pro-inflammatory cytokine release *in vivo* (111–115). Further, low levels might result in the development of inflammatory conditions like allergy, neuronal dysfunction or rheumatoid arthritis (116–118) and potentially cause a shift toward exaggerated Th2 responses (111). Zhang et al. described that pyridoxal and pyridoxal-5-phosphate inhibit the inflammation-associated NLR family pyrin domain containing 3 (NLRP3) inflammasome activation at various levels including cytokine gene expression and caspase processing among other effects like protecting mice against fatal endotoxins (119). Anti-inflammatory mechanisms are primarily based on the active vitamer PLP, which acts as a vital cofactor in the kynurenine pathway of tryptophan degradation, the metabolism of sphingosine 1-phosphate (S1P) and serine hydroxy methyltransferase activity. Moreover, it modulates NF-kB signaling. In contrast, there is evidence of vitamin B6 being compromised in inflammatory conditions like rheumatoid arthritis, as supplementation may improve B6 levels without resolving the underlying inflammatory process, an explanation being the active mobilization to inflammatory sites or its consumption by upregulated metabolic pathways resulting in a drop of hepatic and circulating PLP levels (tissue-specific depletion) due to inflammation (120–122). In fact, vitamin B6 seems to be involved in immunosurveillance via contributing to the sphingosine 1-phosphate metabolism which regulates the lymphocyte migration to the intestine (123, 124). Lastly, PLP has been suggested to mitigate COVID-19 symptom severity (Shakoor et al. (85).

Clinically, Talbott et al. found out that two months supplementation of 50 mg/d pyridoxine hydrochloride in participants aged 65 and older, resembling a standard daily supplementation rather than a high-dose therapeutic intervention, resulted in an elevation of lymphocyte proliferation as a response to B- and T cell mitogens, along with an increased number of T helper cells. These effects were pronounced in individuals with lower initial plasma levels pointing toward the fact that the elderly immune system might benefit from vitamin B6 consumption above intake recommendations (125). Similar results were obtained by Meydani et al., who investigated the immunologic effects of vitamin B6 depletion-repletion on PBMCs isolated from older individuals (126). An analysis of 2229 adults, as part of the Framingham Offspring study, revealed that higher levels of PLP correlate to decreased chronic inflammation and vice versa, as it is involved as a cofactor in various enzymatic reactions as well as in the production of immunomodulatory metabolites (127, 128).

In summary, pyridoxine, its active form PLP respectively, contributes to immunocompetence in the elderly with special regard to lymphocyte development, anti-inflammatory actions and improved immunosurveillance. Supplementation is more beneficial in individuals displaying inadequate B6-levels. Especially in the elderly, pyridoxine deficiency results in an overall compromised immunologic- and pro-inflammatory state with correlating disorders. This highlights the importance to optimize intake levels for geriatric individuals, potentially above current RDAs, to improve physiological resistance to infections. By conducting long-term clinical trials focusing on intracellular biomarkers such as the PAr index (ratio of 4-pyridoxic acid divided by the sum of pyridoxal 5´-phosphate plus pyridoxal (PA:(PLP+PL)) https://bevital.no/par-index/) future research should focus on identifying the interconnection between systemic inflammation and B6 status in the elderly.

## 8 Biotin (vitamin B7): role in immune function and aging

**Figure 7:**
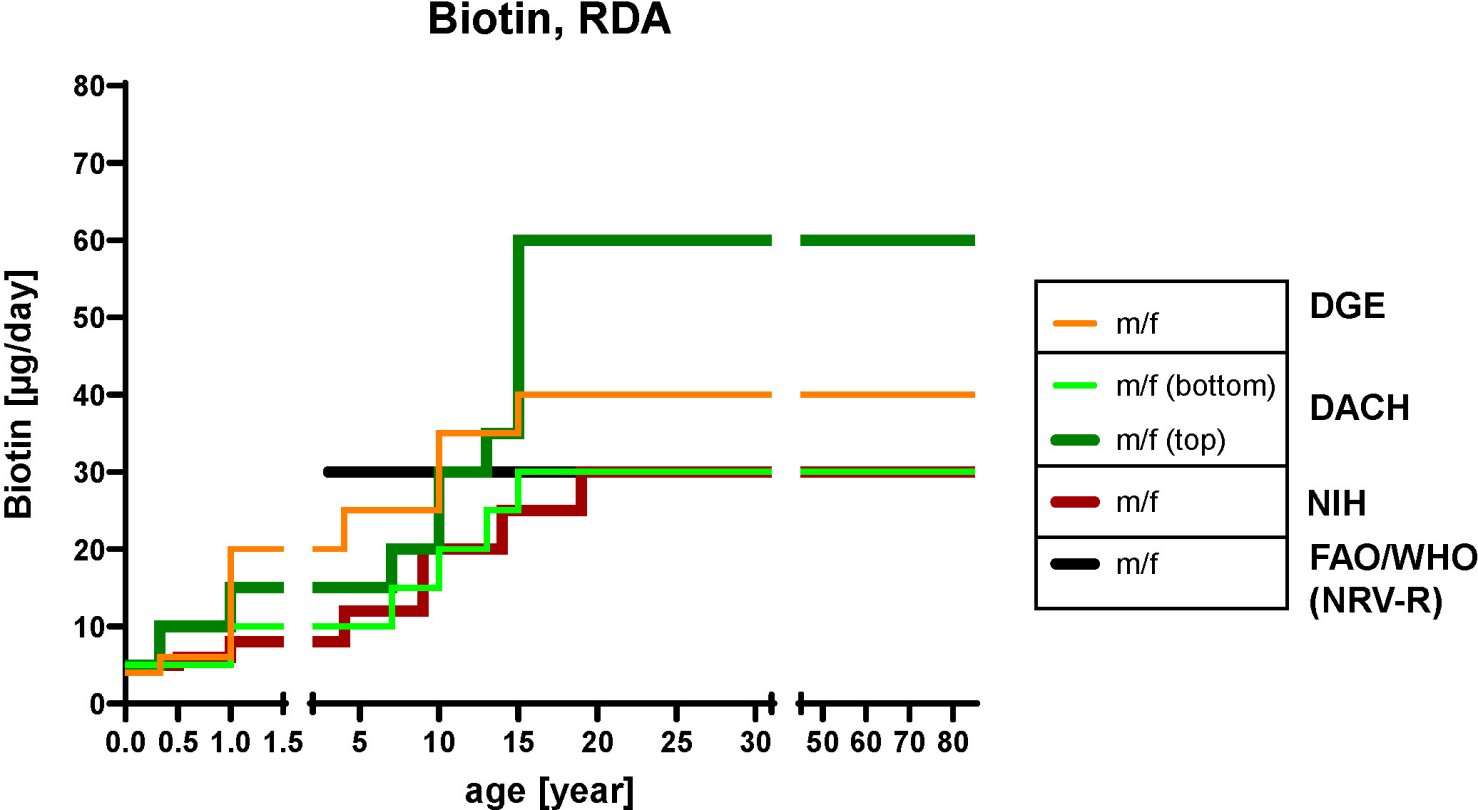
RDA reference values of vitamin B7 (biotin). References according to D-A-CH [German Nutrition Society (DGE); Austrian Nutrition Society (ÖGE); Swiss Nutrition Society (SGE). Referenzwerte für die Nährstoffzufuhr [Dietary Reference Values], 2nd edition, 5th updated issue; German Nutrition Society: Bonn, Germany, 2019; ISBN 9783887492618.], DGE [DGE. Biotin (2025). Available at: https://www.dge.de/wissenschaft/referenzwerte/biotin/ (Accessed May 26, 2025). (131)], NIH [Institute of Medicine. Dietary Reference Intakes for Thiamin, Riboflavin, Niacin, Vitamin B6, Folate, Vitamin B12, Pantothenic Acid, Biotin, and Choline. Washington: National Academies Press (2000) (38)] and the NRVs-R of FAO/WHO [Lewis J. Codex nutient reference values: Especially for vitamins, minerals and protein, p. 36. Rome. FAO and WHO (2019) (39)]. D-A-CH, Deutschland, Austria, Confoederatio Helvetica (eng. GSA, Germany, Switzerland, Austria); DGE, Deutsche Gesellschaft für Ernährung (eng. German Nutrition Society); FAO, Food and Agriculture Organization; NIH, National Institutes of Health; NRV-R, Nutrient Reference Value-Requirement; ÖGE, Österreichische Gesellschaft für Ernährung RDA, Recommended Dietary Allowance; SGE, Schweizerische Gesellschaft für Ernährung WHO, World Health Organization.

Vitamin B7 (**Figure 1E**), also known as biotin, predominantly acts as a (carboxylase)-cofactor in fatty acid-, glucose- and amino acid metabolism, hence energy production (129) but it seems to play a role in chronic inflammation as well (130). **Figure 7** as well as **Table 1** illustrate the RDA for vitamin B7 according to the different health institutions with recommendations for the elderly as well.

Biotin deficiency can be caused by insufficient dietary intake of beans, milk, soy, oilseed and nuts or the challenging absorption of the vitamin that is produced by bacteria in the colon. Deficiency is also triggered by the consumption of antinutrients such as avidin from raw egg-white and results in seizures, alopecia, neurological conditions and, in the context of immunity, altered transcription factor expression such as NF-κB or specificity proteins 1 and 3 (SP1/3), among others (20, 132–137). Research regarding CD4+ T lymphocytes revealed that biotin deficiency triggers mechanistic target of rapamycin (mTOR) signaling which enhances Th1- and Th17-induced pro-inflammatory responses (138). Additionally, Sakurai-Yageta and Suzuki comprehensively examined how biotin deficiency contributes to inflammatory processes and how it generally supplies a normal functioning immune system status (139). Due to the fact, that the water-soluble vitamin consumed via food is mostly peptide- or lysine-bound and therefore needs to be released via pancreatic biotinidase (140), the latter enzyme might be also important regarding potential deficiencies (133).

General immunologic impacts of vitamin B7-consumption include anti-inflammatory reactions as well as the regulation of immune-system related processes involving T cell cytotoxicity or susceptibility to infections (141, 142). Kuroishi et al. demonstrated that low levels of biotin exacerbate allergic reactions to nickel in mice, along with an increased production of IL-1β suggesting that intake of the vitamin might be of interest in the context of inflammation-associated metal allergies in humans as well (132, 143). Moreover, vitamin B7 binding to biotinylating histones results in a diminished NF-κB gene expression (144–146). In turn, low levels of vitamin B7 correspond to a stunted activation of AMP kinase in human monocyte-derived dendritic cells, which enhances pro-inflammatory reactions such as the release of associated cytokines like, e.g., TNF-α, IL-12p40, IL-23 and IL-1β (133).

However, only a limited number of *in-vivo* studies and particularly RCTs explore the immune-boosting effects of biotin on older individuals. Generally, the process of aging per se does not seem to affect serum biotin levels but varies greatly among aged individuals (147).

In summary, biotin status among the elderly varies interindividually, resulting in the necessity for future research to potentially revise dietary or supplemental intake recommendations regarding optimal immune protection in the elderly, as vitamin B7 plays an important role in, e.g., epigenetic modulations and inflammatory signaling. RCTs but also pre-clinical research regarding the immunomodulative capacity of biotin are scarce. Relevant insights comprise its involvement in the suppression of chronic inflammation or vice versa its role in activating pro-inflammatory pathways upon deficiency, which is relevant for “inflammaging”. Hence, human (high-dose) intervention studies need to be conducted to gain further insight into the interplay of biotin and the immune system and its therapeutic potential, examples being vaccination efficacy, immune cell development or autoimmune disorders.

## 9 Folates (vitamin B9): role in immune function and aging

Vitamin B9, also known as folate or folic acid (**Figure 1G**), exhibits various biochemical functions including one-carbon transfer reactions (tetrahydrofolate (THF)), DNA repair, synthesis and methylation, breakdown and synthesis of neurotransmitters, e.g., norepinephrine, dopamine as well as serotonin (5-methyl tetrahydrofolate 5-MTHF and the recycling of homocysteine into methionine together with vitamin B6 and B12 (methylation reactions), just to name a few (148–151). Folate and vitamin B12 are dependent on each other, as Partearroyo et al. demonstrated (152). It seems that the ratio of B9 and B12 (balanced intake) is comparably important as their absolute dietary concentrations. An induced imbalance resulted in, e.g., alteration of NK cell-mediated cytotoxicity in aged rats (152). The vitamin needs to be supplied via diet (juices, dark leafy green vegetables, fortified foods or citrus fruits) or supplements respectively (71, 153).

**Figure 8:**
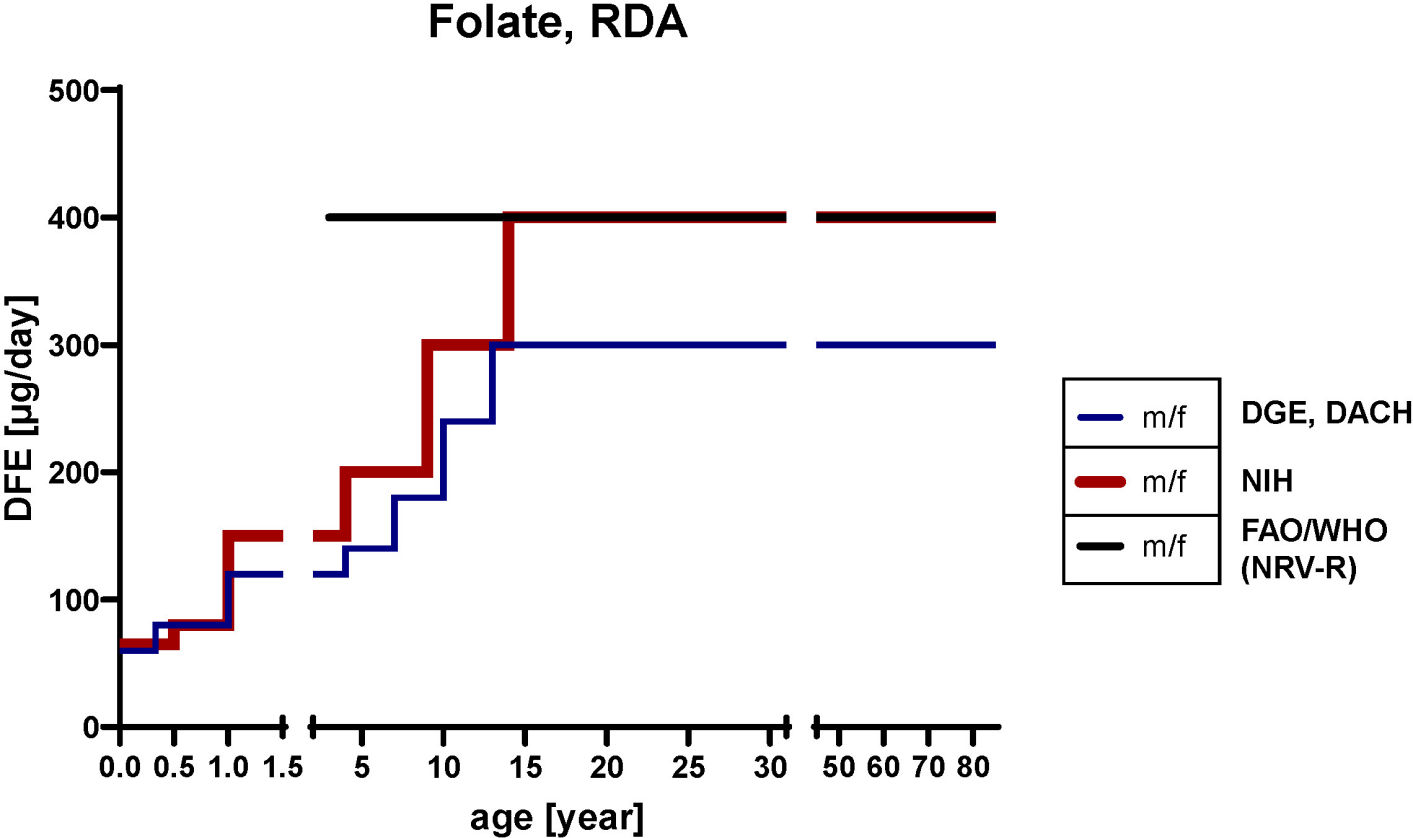
RDA reference values of vitamin B9 (folate). References according to D-A-CH [German Nutrition Society (DGE); Austrian Nutrition Society (ÖGE); Swiss Nutrition Society (SGE). Referenzwerte für die Nährstoffzufuhr [Dietary Reference Values], 2nd edition, 5th updated issue; German Nutrition Society: Bonn, Germany, 2019; ISBN 9783887492618.], DGE [DGE. Folat (2025). Available at: https://www.dge.de/wissenschaft/referenzwerte/folat/ (Accessed May 26, 2025). (45)], NIH [Institute of Medicine. Dietary Reference Intakes for Thiamin, Riboflavin, Niacin, Vitamin B6, Folate, Vitamin B12, Pantothenic Acid, Biotin, and Choline. Washington: National Academies Press (2000) (38)] and the NRVs-R of FAO/WHO [Lewis J. Codex nutient reference values: Especially for vitamins, minerals and protein, p. 36. Rome. FAO and WHO (2019) (39)]. D-A-CH, Deutschland, Austria, Confoederatio Helvetica (eng. GSA, Germany, Switzerland, Austria); DFE, Dietary Folate Equivalent, DGE, Deutsche Gesellschaft für Ernährung (eng. German Nutrition Society); FAO, Food and Agriculture Organization; NIH, National Institutes of Health; NRV-R, Nutrient Reference Value-Requirement; ÖGE, Österreichische Gesellschaft für Ernährung RDA, Recommended Dietary Allowance; SGE, Schweizerische Gesellschaft für Ernährung WHO, World Health Organization.

**Figure 8** as well as **Table 1** illustrate the RDA for vitamin B9 according to the different health institutions with recommendations for the elderly as well. In turn, folic acid deficiency is quite common [**Supplementary Figure 5**; percentage of people not having an adequate supply (around 90% of people aged 65 and older below D-A-CH reference levels) based on data of the German National Nutrition Survey II (2005–2007) (154)] and results in serious conditions comprising shortness of breath, depression, weakness, cardiovascular diseases, fatigue, elevated homocysteine levels which leads to systemic and vascular inflammation, anemia, dementia or Alzheimer’s disease among others (150, 155, 156).

Immunologic consequences comprise a reduced CD4+/CD8+ T cell ratio following the diminished numbers of cytotoxic CD8+ T cells, together with an overall decrease in the proportion of T cells as well as their activation upon mitogen activation, resulting in a deteriorated protection against infections (157, 158). Other implications might involve impaired DTH response or decreased antibody response (18, 159, 160).

Folic acid participates in cell-mediated immunity, exceeding its participation in the humoral immunity, examples being the increased proliferation of cytotoxic CD8+ T cells next to the elevated expression of anti-apoptotic B-cell lymphoma 2 (Bcl-2) protein leading to the pronounced survival of T cells as Treg cells which consecutively express the folate receptor 4. Therefore, low folate intake decreases Tregs-numbers in the intestines, resulting in increased susceptibility concerning intestinal inflammatory responses (157, 161, 162). Supplementation exhibits positive immunologic impacts ranging from decreased susceptibility toward infections, over delayed hypersensitivity responses, increased phagocytosis, immunoglobulin production and innate immunity in older people, improved Th1 response, up to noteworthy effects on T lymphocyte proliferation and blastogenic responses (71, 163, 164). Field et al. investigated older vs. younger male rats and point toward the importance of an increased dietary or supplementary folate intake in the elderly to counteract ageing-associated immunologic changes and to enhance parameters like proliferative response to mitogens or cytokine production in the spleen (165).

Regarding the clinical relevance, a study by Troen et al. involving 105 healthy, postmenopausal women (age 50–70) revealed, that folic acid supplementation might benefit those having a low dietary vitamin B9 intake, whereas excessive intake-especially in combination with a folate-rich diet-potentially suppresses immune function in the elderly, manifesting as reduced NK cell cytotoxicity, likely due to unmetabolized folic acid (163). Contrary to that, a study conducted by Bunout et al. found that a four month-supplementation regimen (400 µg folic acid among other nutrients in addition to the regular diet) in healthy people aged 70 and over, resulted in an increase in NK cell cytotoxicity as well as fewer infection rates (164). Recently, there have been reports of folic acid playing a protective role in the early stages of COVID-19-associated respiratory disease by inhibiting furin, a bacterial-and viral infection-associated enzyme, concomitantly inhibiting its binding by SARS-CoV-2 spike protein, cell entry and therefore virus turnover (166). In close proximity to that, folic acid, THF and 5-MTHF have been shown to exhibit prominent binding affinities against SARS-CoV-2, highlighting its potential role in the treatment of COVID-19 (167).

In conclusion, folate plays a critical role in the immune system of the elderly, specifically targeting cell-mediated immunity while concomitantly enhancing the ageing individual’s response to infections. However, a discrepancy remains between an increased demand with age, a prominent undersupply and potentially negative immunologic impacts upon overconsumption like suppressed NK cell function. As a result, standardized clinical trials involving geriatric cohorts with the focus on identifying dose-response interactions and the ideal intake recommendations combining the positive immunological impacts of an adequate supply status while preventing inverse outcomes due to supraphysiological folate levels to induce immunological resilience need to be conducted. Vitamin B9 and vitamin B12 interplay is of great scientific interest concerning exacerbated immune defects. Moreover, genetic variants such as the MTHFR C677T genotype could also be considered in future research projects with regard to immunosenescent-, and age-related alterations in the biological activity of folate.

## 10 Cobalamin (vitamin B12): role in immune function and aging

In recent years, vitamin B12 (**Figure 1I**), also known as cyanocobalamin (metabolically active form), methylcobalamin, cob(I)alamin, 5′-deoxyadenosylcobalamin or hydroxycobalamin (105), has become of interest regarding general public health concerns and immunologic aspects in particular. As already depicted in the folate chapter, folate and cobalamin are in close functional proximity as they are mutually dependent on each other for activation. B12 and its analogs perform a variety of important biochemical functions including the regulation of the nervous system, an example being nerve cell maintenance, DNA synthesis, hematopoiesis or fatty acid- as well as amino acid metabolism (105, 168). With regard, the human body unfortunately cannot absorb the vitamin B12 being synthesized by intestinal bacteria (colon). Therefore, the ingestion of mainly animal derived products (cobalamin content dependent on biomagnification processes through food chains), nutritional yeast, fortified foods or supplements are necessary to meet the intake recommendations (164, 169).

**Figure 9:**
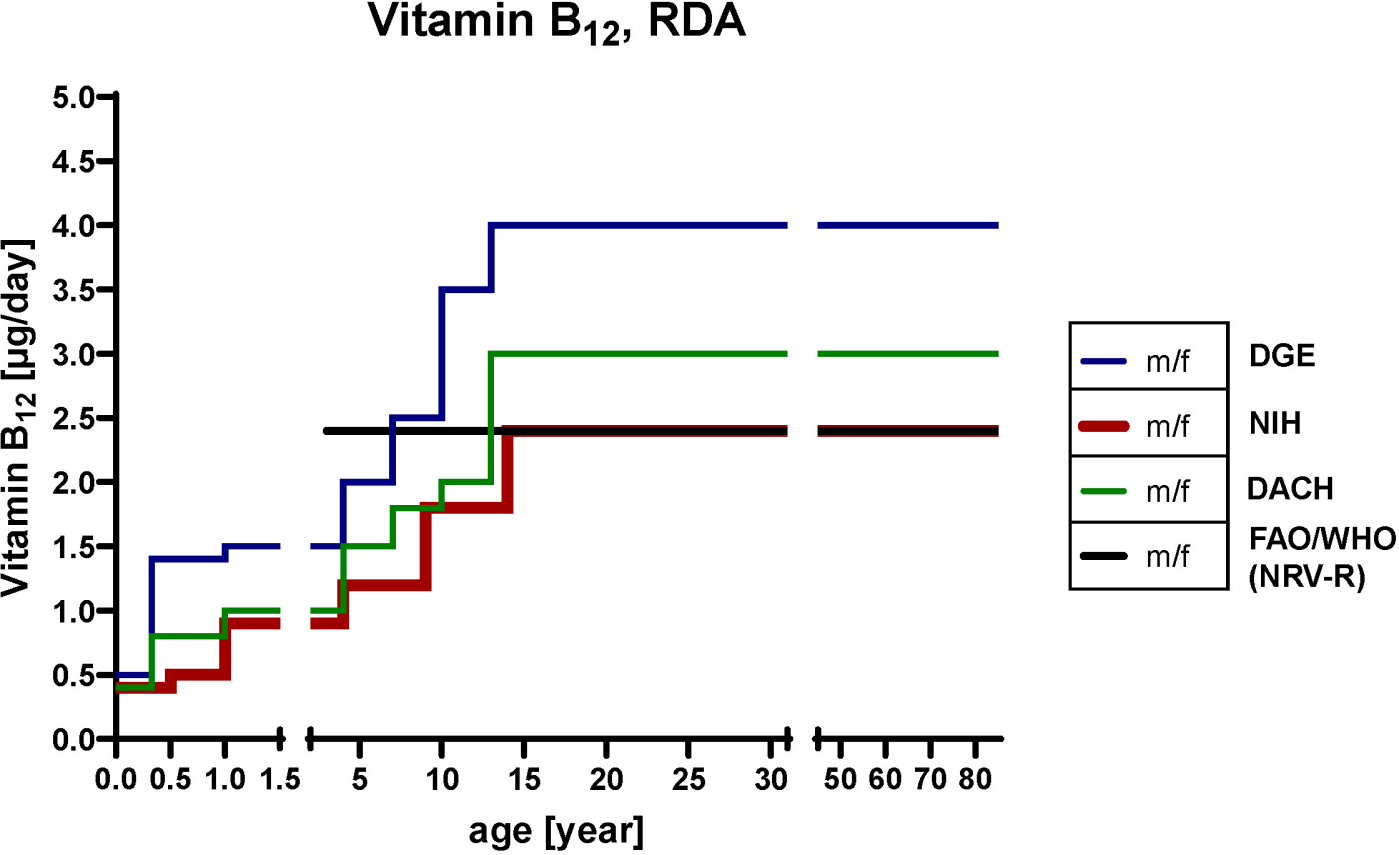
RDA reference values of vitamin B12. References according to D-A-CH [German Nutrition Society (DGE); Austrian Nutrition Society (ÖGE); Swiss Nutrition Society (SGE). Referenzwerte für die Nährstoffzufuhr [Dietary Reference Values], 2nd edition, 5th updated issue; German Nutrition Society: Bonn, Germany, 2019; ISBN 9783887492618.], DGE [DGE. vitamin B_12_ (Cobalamine) (2025) (cited 2025 May 26). Available from: https://www.dge.de/wissenschaft/referenzwerte/vitamin-b12/ (Accessed May 26, 2025). (170)], NIH [Institute of Medicine. Dietary Reference Intakes for Thiamin, Riboflavin, Niacin, Vitamin B6, Folate, Vitamin B12, Pantothenic Acid, Biotin, and Choline. Washington: National Academies Press (2000) (38)] and the NRVs-R of FAO/WHO [Lewis J. Codex nutient reference values: Especially for vitamins, minerals and protein, p. 36. Rome: FAO and WHO (2019) (39)]. D-A-CH, Deutschland, Austria, Confoederatio Helvetica (eng. GSA, Germany, Switzerland, Austria); DGE, Deutsche Gesellschaft für Ernährung (eng. German Nutrition Society); FAO, Food and Agriculture Organization; NIH, National Institutes of Health; NRV-R, Nutrient Reference Value-Requirement; ÖGE, Österreichische Gesellschaft für Ernährung RDA, Recommended Dietary Allowance; SGE, Schweizerische Gesellschaft für Ernährung WHO, World Health Organization.

**Figure 9** as well as **Table 1** illustrate the RDA for vitamin B12 according to the different health institutions with recommendations for the elderly as well. Due to aging-related challenges like inadequate dietary consumption, disregard of elevated intake recommendations, insufficient intrinsic factor production by parietal cells within the stomach or malabsorption, the elderly represent one of the many population groups that is frequently affected by vitamin B12 deficiency. This might be partially due to the process of inflammaging which results in a B12 deprivation in, e.g., macrophages (171).

**Supplementary Figure 6** shows the percentage of people not having an adequate supply (around 10–30% of people aged 65 and older below D-A-CH reference levels) based on data of the German National Nutrition Survey II (2005–2007) (172). Common pathologic deficiency symptoms might involve peripheral tingling, fatigue, weight loss, loss of appetite, pernicious anemia and DNA production impairment due to decreased folate activation (less biologically active 5-methyltetrahydrofolate).

Interestingly, immunologic consequences such as hampered immune cell functioning, increased levels of macrophage-derived TNF-α and IL-6 or the reduced synthesis of CD8+ (increased CD4+/CD8+ ratio)- and NK cells (105, 152, 173, 174). In accordance, low vitamin B12 levels might result in lymphocyte downregulation as well as loss of NK cell functionality which seems to be reversible upon therapeutic B12 administration (174). A proper vitamin B12 status seems to be of great importance when it comes to the (ageing-associated) cell-mediated rather than humoral immune system (105).

Clinically, B12 appears to be important for the innate immune system, namely increased NK cell cytotoxicity, as Bunout et al. observed after supplementing healthy individuals aged 70 and older with 400 µg folic acid and 3.8 µg B12, among other micronutrients, over the course of four months (164). Moreover, low vitamin B12 serum levels seem to impair pneumococcal polysaccharide vaccine antibody response in immunocompetent older individuals (65+ y/o) (175).

In conclusion, an adequate vitamin B12 status significantly contributes to the aging immune system by enhancing immunocompetence via increasing cell-mediated immunity and cytotoxic effector cells in particular. Diminished cobalamin levels on the other hand, which are prevalent among the elderly, contribute to inflammaging. In order to investigate vitamin B12’s potential to restore immune resilience within the geriatric population and pinpoint anti-inflammatory actions, RCTs determining functional biomarkers such as holotranscobalamin and methylmalonic acid need to answer the research question how targeted B12 supplementation affects the senescence-associated secretory phenotype (SASP), next to reducing the current knowledge gaps regarding optimized dose-response relationships and vitamin B9-B12-imbalances in the elderly.

## 11 Ascorbate (vitamin C): role in immune function and aging

**Figure 10:**
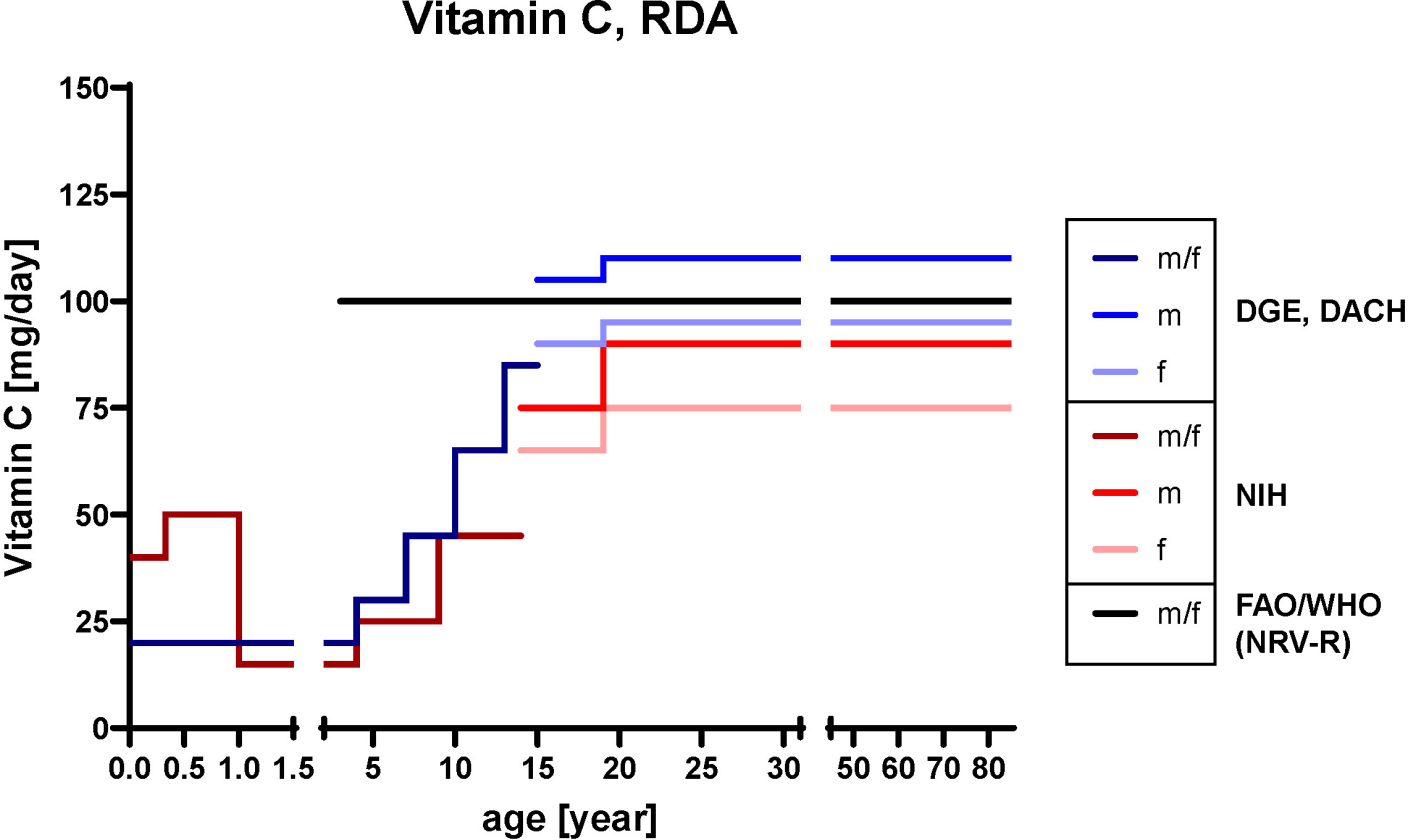
RDA reference values of vitamin C. References according to D-A-CH [German Nutrition Society (DGE); Austrian Nutrition Society (ÖGE); Swiss Nutrition Society (SGE). Referenzwerte für die Nährstoffzufuhr [Dietary Reference Values], 2nd edition, 5th updated issue; German Nutrition Society: Bonn, Germany, 2019; ISBN 9783887492618., DGE. Vitamin C (2025). Available from: https://www.dge.de/wissenschaft/referenzwerte/vitamin-c/ (Accessed May 26, 2025). (179)], NIH [Institute of Medicine. Dietary Reference Intakes for Vitamin C, Vitamin E, Selenium, and Carotenoids. Washington: National Academies Press (2000) (180)] and the NRVs-R of FAO/WHO [Lewis J. Codex nutient reference values: Especially for vitamins, minerals and protein, p. 57. Rome: FAO and WHO (2019) (39)]. D-A-CH, Deutschland, Austria, Confoederatio Helvetica (eng. GSA, Germany, Switzerland, Austria); DGE, Deutsche Gesellschaft für Ernährung (eng. German Nutrition Society); FAO, Food and Agriculture Organization; NIH, National Institutes of Health; NRV-R, Nutrient Reference Value-Requirement; ÖGE, Österreichische Gesellschaft für Ernährung RDA, Recommended Dietary Allowance; SGE, Schweizerische Gesellschaft für Ernährung WHO, World Health Organization.

Vitamin C (**Figure 1H**), also known as ascorbic acid, is a crucial micronutrient. Unlike many mammals, humans are not able to synthesize vitamin C and require regular vitamin C intake (176, 177). The optimal serum levels for vitamin C range between 30–90 µmol/L, while concentrations below 11 µmol/L represent a deficiency (178). **Figure 10** as well as **Table 1** illustrate the RDA for vitamin C according to the different health institutions with recommendations for the elderly as well. The recommended dietary intake of vitamin C for adults is 65–90 mg (about 90 mg/day vitamin C for men and 75 mg/day for women) (180, 181), which supports immune homeostasis and metabolic functions (182). Supplementing with 100 mg per day can help to saturate blood levels of vitamin C which in turn seems to be important to reduce the risk of, e.g., heart disease, stroke and pancreatic cancer (181, 183). Vitamin C plays an essential role as a cofactor for enzymes, and is involved in collagen synthesis, neurotransmitter production, and enhancing non-heme iron absorption (181, 184–186). It also acts as a potent antioxidant, protecting the body against oxidative stress by neutralizing free radicals, other ROS, and DNA mutations induced by oxidative stress (187, 188).

Johnston et al. (189) indicate the correlation between vitamin C deficiency and an increased susceptibility to severe respiratory infections. Moreover, **Supplementary Figure 7** shows the percentage of people not having an adequate supply (around 30% of people aged 65 and older below D-A-CH reference levels) based on data of the German National Nutrition Survey II (2005–2007) (190).

Regarding the immunomodulatory effects of vitamin C, white blood cells, such as neutrophils, monocytes, leukocytes and lymphocytes, actively accumulate the vitamin in concentrations far higher than in plasma, highlighting its functional role in immunity (191–194). This helps protecting immune cells from the damaging effects of ROS produced during inflammation (195). Latest investigations examined the inhibitory effects of vitamin C on the expression of pro-inflammatory mediators like IL-6 and TNF-α and revealed how vitamin C prevents excessive immune responses (182). The supplementation of vitamin C improves immune response mediated by lymphocytes, lymphocyte proliferation, antimicrobial and NK cell activity, chemotaxis, production of cytotoxic T cells and DTH reactions (19, 196–202). Phagocytic cells like neutrophils and macrophages store high levels of vitamin C, which enhances their ability to migrate to infection sites, phagocytize microbes and generate oxidants for microbial killing (203–205). Jeng et al. showed that supplementing vitamin C (1–3 g/day) improved neutrophil function in healthy adults (206). Additionally, it helps regulating the immune response by promoting neutrophil apoptosis, preventing excessive immune activation and reducing tissue damage (207). Antibody production is also enhanced by vitamin C. High doses of 1000 mg/day for 75 days significantly increased levels of IgA, IgG and IgM antibodies, offering stronger protection against infections and cancerous cells (208). Research has shown that vitamin C can promote NK-cell proliferation, even aiding the expansion of NK progenitors from stem cells when cultured with cytokines (209). Mousavi et al. summarized that vitamin C inhibits bacterial growth by inducing oxidative stress in certain bacterial species (210). Vitamin C is also vital for wound healing, facilitating processes like fibroblast migration and neovascularization through its impact on collagen formation and plays a protective role by preventing lipid peroxidation and scavenging free radicals (211, 212). Though its effects on allergic and inflammatory diseases are not fully understood, high-dose vitamin C may modulate the immune response through inhibition of the NF-κB signaling pathway, with p38 MAPK proposed as a target (213).

Clinically, various studies have shown that both aging and vitamin C deficiency lead to immune defects (198). Aging is associated to reduced humoral-mediated and cellular-mediated immunity (214). In addition, several studies revealed the immune-modulating and immune-stimulating effect of vitamin C by influencing both the innate and adaptive immune responses (215, 216). Compared to younger adults, the elderly tend to have lower circulating vitamin C levels, which impacts immune cell function (217, 218). These aberrations may contribute to higher susceptibility to infection and diseases, low serum and tissue levels of vitamin C, increasing oxidative stress and inflammation (198, 219). The immune response of elderly is impaired, promoting the development of many diseases such as neurodegenerative disease, cancer and others (198, 219, 220). As reviewed by Wintergerst et al. a reduced vitamin C concentration in the elderly appears to be an indicator of all-cause mortality and mortality from cardiovascular diseases (221). However, there is limited evidence that healthy aging leads to lower levels or higher requirements of vitamin C (222). Hospitalized patients, especially in older patient populations, have lower vitamin C status and enhanced vitamin C requirements than the general population (223), mainly through low intakes or chronic illnesses (222). Noteworthy, elderly hospitalized patients with acute respiratory infections have shown a significantly better outcome than those not receiving the vitamin (224). However, vitamin C can help restoring immune function in the elderly. Lymphocytes from older adults pre-treated with vitamin C (physiological extracellular plasma concentration of 10 µg/ml) showed restored proliferation to youthful levels (198). Zychowska et al. hypothesized that vitamin C supplementation might mitigate inflammaging accompanied by chronic inflammation and prolonged oxidative stress, therefore being potentially beneficial for older people subjected to physical activity (220). In a placebo-controlled study, the intramuscular administration of 500 mg/day vitamin C improved T-cell proliferation in elderly participants, highlighting its immune-boosting potential (198). Vitamin C supplementation can enhance NK-cell activity, helping them target and kill tumor cells by reducing the protective effect of platelets that shield cancer cells. This effect could potentially help prevent cancer metastasis (208). Vitamin C also enhanced T-cell response in a placebo-controlled trial but neither altered serum immunoglobulin levels (IgA, IgM and IgG) nor the proportion of E-rosette-forming cells (lymphocytes with three or more adherent sheep red blood cells) (198). Delafuente et al. investigated the effects of vitamin C (2 g/day/oral intake) on both *in vitro* and *in vivo* immunologic parameters in older adults (214). The three weeks of treatment did not affect the immune defense, which is probably due to a treatment duration that is too short compared to, e.g., Andrews et al., who demonstrated that nine months of vitamin C treatment in an elderly population were necessary to replete vitamin C stores equal to those of young people, an explanation being that the elderly seem to be storing vitamin C less efficiently next to potential age-related alterations in pharmacodynamic and pharmacokinetic parameters (214, 225). Interestingly and in contrast to earlier assumptions, a study on the depletion and repletion kinetics of vitamin C conducted by Blanchard (226) indicated that there are no significant differences between younger and older adults (226). Moreover, data suggests that a daily dose of approximately 200 mg of ascorbate from fruits and vegetables is beneficial concerning plasma and immune cell saturation as well as maximizing bioavailability (227). Lymphocytes rely on vitamin C to enhance their proliferation in response to threats. This proliferative response has been observed to improve on vitamin C supplementation, especially in older adults and aging laboratory animals (197, 215).

In conclusion, ascorbate significantly contributes to a properly maintained immune status in the elderly. Vitamin C’s immunomodulatory potential involves potent antioxidative activities like counteracting oxidative damage by actively accumulating the vitamin in leukocytes and conveying anti-inflammatory responses regarding cell-mediated immunity, thereby directly impacting the inflammaging phenotype. Low serum levels suggesting physiological undersupply seem particularly relevant for the hospitalized elderly as critically depleted stores correlate with an increased susceptibility to severe respiratory tract infections. However, optimal repletion periods, dosages and saturation levels to achieve clinical relevance in the elderly are yet to be determined. Hence, large-scale human trials and metabolomic approaches need to be prioritized in the future in order to identify vitamin C’s involvement in counteracting immunosenescence and improving clinical endpoints like enhanced protection against infections.

## 12 Discussion – conclusion and future aspects

Aging is associated with a gradual deterioration of the innate and adaptive immune system often in conjunction with chronic low-grade inflammation. Therefore, terms such as “immune senescence” and “inflammaging” were introduced. A well-balanced diet and micronutrient supplementation supposedly counteract immunologic deterioration while micronutrient deficiency of different water-soluble vitamins is suggested to enhance susceptibility to common infections by various studies described in the respective chapters.

Vitamin B1 is, e.g., involved in the thymic differentiation of T cells as well as energy metabolism of immune cells and immunometabolism in general, highlighting its possible importance regarding immunocompetence and overall health in the elderly, especially when vitamin supply recommendations are not met. RCTs or human trials concerning thiamine directly influencing immune parameters, especially regarding the elderly, are scarce, whereas there are clear indications of anti-inflammatory effects.

Vitamin B2 counteracts the release of pro-inflammatory cytokines and increased neutrophil, monocyte and macrophage numbers *in vivo* but >41% of free-living elderly people were reported to not meet the recommended vitamin B2-supply (61, 62; Mikkelsen and Apostolopoulos) n

Vitamin B3 is, e.g., important for lipid metabolism and exhibited anti-inflammatory properties and induced Treg differentiation (82) but also decreased monocyte chemotaxis and pro-inflammatory cytokines among others (77–81). Pronounced effects on innate immune response were described (80, 83, 84). Inadequate supply occurs less frequently than with thiamine and riboflavin.

Vitamin B5 with its important role in CoA synthesis contributes to immune responses by elevating levels of acute-phase proteins (89, 90). The broad immunomodulatory spectrum contains enhancement of CD8+ cytotoxic T cell differentiation and anticancer immunosurveillance (96) but also Th1- and Th17 cell differentiation and macrophage maturation among others (100–103). Even though deficiency is not very common, there are clear indications of a generally higher susceptibility to infections when a deficiency is present.

Vitamin B6 has many immune system associated functions such as differentiation, proliferation and maturation of lymphocytes and high levels of PLP exhibit also anti-inflammatory effects (19, 107, 127, 128, 202, 228). In aged patients, lymphocyte proliferation was induced after supplementation with high levels (50 mg/d) (125). Vitamin B6 affects also antibody production, NK-cell activity, T-cell response and cytokine release (111–115) also with strong effects on lymphocyte migration to the intestines (123, 124).

For the immune-stimulating effects of vitamin B7, there is also only a very limited number of *in vivo* studies available. Insufficient levels result in human monocyte-derived dendritic activation and, therefore, pro-inflammatory reactions but also Th1- and Th17-induced pro-inflammatory responses (133, 138).

Vitamin B9 and B12 are closely related due to the close networking and overlap of their biochemical pathways. Many effects on immune function were described, such as decreased susceptibility toward infections, delayed hypersensitivity responses, increased phagocytosis or improved immunoglobulin production. Moreover, many studies describe a positive impact on innate immunity in older people, NK cell cytotoxicity and Th1 responses next to beneficial effects on T lymphocyte proliferation (increased T cell and Treg survival and proliferation of CD8+ cells as well as blastogenic responses) (71, 157, 161, 163, 164). Noteworthy, excessive B9 intake combined with a folate-rich diet could suppress immune function in the elderly (163). 10–30% of people aged 65 and older do not have an adequate B12 supply.

Vitamin C seems to protect immune cells due to its pronounced antioxidant properties. Different types of immune cells contain high intracellular levels of this vitamin and it exhibits protective properties. While it shows immunomodulatory and immunostimulating effects, a deficiency is clearly associated with higher susceptibility to infections. Hospitalized patients with low plasma levels are also suggested to have worse outcomes compared to patients at good supply. The effects of vitamin C are broad such as improvement of the immune response of the innate and adaptive immune system and increased lymphocyte proliferation, including NK cell activity.

In summary, many *in vitro*- and *in vivo* studies point towards an immunomodulatory function of distinct water-soluble vitamins. Overall, the number of available epidemiological studies - and in particular interventional studies, especially RCTs - on B vitamins in the context of an aging population is surprisingly limited. While there are significantly more human studies on vitamin C in the context of the immune system, even here, studies rarely focus on the aging population or on potential interventions to strengthen their immune system. Particularly in the case of interventional studies for water-soluble vitamins, the cohort size is often rather small, and it must be taken into account that the definition of “older adults” is not uniform and that there are significant differences between healthy older adults and those with multiple chronic conditions, which further drastically reduce the comparability of such studies. Furthermore, rather than using single-vitamin supplements - as would be the case in drug trials - multivitamin supplements and other combinations of micronutrients are very often used, which makes it even more difficult to isolate the specific effects of individual vitamins, especially given the often small sample sizes. Moreover, the status of individual vitamin intake is often poorly assessed at the start of the study, and since many foods today are specifically fortified with vitamins, supplementation that the subject or patient is unaware of is a frequent issue. Significant effects are also, in principle, more difficult to demonstrate, as even the control groups always have a baseline vitamin intake that is often subject to large interindividual variations. All of this leads to significant gaps in knowledge that can only be resolved through consistent standardization of the definitions of the aging population itself, as well as the classification of healthy older adults and older patients. In addition, awareness must be raised that the ever-growing aging population could be treated cost-effectively if research into interventions and prevention using micronutrients were better supported by appropriate human studies with large sample sizes. It is noteworthy, however, that anti-inflammatory effects can be demonstrated for all water-soluble micronutrients, although the necessary dosage is often not clarified.

Therefore, this review aimed to summarize the current state of immunologic research in the context of water-soluble vitamins and the aging population and, to that end, to draw the attention of scientists and clinicians to the significant gaps in our current knowledge and to raise their awareness of the great potential, the very favorable risk-benefit profile, and, not least, the low costs associated with providing adequate micronutrient supply for an increasingly aging population. It stands to reason that these population groups could also benefit from supplementation in terms of protection against common infectious diseases, especially if a balanced and varied diet cannot be fully realized. It is also important to critically re-evaluate the recommended intake levels for vitamins in older patients with very different clinical presentations.

## Supplementary material

Supplementary Figure 1 Results of the German National Nutrition Survey II (2005–2007) [Max Rubner-Institut. Nationale Verzehrsstudie II. Ergebnisbericht, Teil 2: Die bundesweite Befragung zur Ernährung von Jugendlichen und Erwachsenen. p. 250. MRI, Karlsruhe, Germany (2008). Available at: https://www.openagrar.de/receive/bmelv_mods_00000135 (Accessed February 24, 2026) (46)]. Percentage of people [male (m), n = 7093; female (f), n = 8278] of different ages who do not reach the recommended vitamin B1 intake according to D-A-CH reference values [German Nutrition Society (DGE); Austrian Nutrition Society (ÖGE); Swiss Nutrition Society (SGE). Referenzwerte für die Nährstoffzufuhr [Dietary Reference Values], 2nd edition, 5th updated issue; German Nutrition Society: Bonn, Germany, 2019; ISBN 9783887492618.]. Mean values are represented as dotted lines. D-A-CH, Deutschland, Austria, Confoederatio Helvetica (eng. GSA, Germany, Switzerland, Austria) DGE, Deutsche Gesellschaft für Ernährung; ÖGE, Österreichische Gesellschaft für Ernährung; SGE, Schweizerische Gesellschaft für Ernährung..

Supplementary Figure 2 Results of the German National Nutrition Survey II (2005–2007) [Max Rubner-Institut. Nationale Verzehrsstudie II. Ergebnisbericht, Teil 2: Die bundesweite Befragung zur Ernährung von Jugendlichen und Erwachsenen. p. 251. MRI, Karlsruhe, Germany (2008). Available at: https://www.openagrar.de/receive/bmelv_mods_00000135 (Accessed February 24, 2026) (64)]. Percentage of people (male (m), n = 7093; female (f), n = 8278) of different ages who do not reach the recommended vitamin B2 intake according to D-A-CH reference values [German Nutrition Society (DGE); Austrian Nutrition Society (ÖGE); Swiss Nutrition Society (SGE). Referenzwerte für die Nährstoffzufuhr [Dietary Reference Values], 2nd edition, 5th updated issue; German Nutrition Society: Bonn, Germany, 2019; ISBN 9783887492618.]. Mean values are represented as dotted lines. D-A-CH, Deutschland, Austria, Confoederatio Helvetica (eng. GSA, Germany, Switzerland, Austria). DGE, Deutsche Gesellschaft für Ernährung; ÖGE, Österreichische Gesellschaft für Ernährung; SGE, Schweizerische Gesellschaft für Ernährung.

Supplementary Figure 3 Results of the German National Nutrition Survey II (2005–2007) [Max Rubner-Institut. Nationale Verzehrsstudie II. Ergebnisbericht, Teil 2: Die bundesweite Befragung zur Ernährung von Jugendlichen und Erwachsenen. p. 252. MRI, Karlsruhe, Germany (2008). Available at: https://www.openagrar.de/receive/bmelv_mods_00000135 (Accessed February 24, 2026) (76)]. Percentage of people (male (m), n = 7093; female (f), n = 8278) of different ages who do not reach the recommended vitamin B3 (niacin) intake according to D-A-CH reference values [German Nutrition Society (DGE); Austrian Nutrition Society (ÖGE); Swiss Nutrition Society (SGE). Referenzwerte für die Nährstoffzufuhr [Dietary Reference Values], 2nd edition, 5th updated issue; German Nutrition Society: Bonn, Germany, 2019; ISBN 9783887492618.]. Mean values are represented as dotted lines. D-A-CH, Deutschland, Austria, Confoederatio Helvetica (eng. GSA, Germany, Switzerland, Austria) DGE, Deutsche Gesellschaft für Ernährung; ÖGE, Österreichische Gesellschaft für Ernährung; SGE, Schweizerische Gesellschaft für Ernährung.

Supplementary Figure 4 Results of the German National Nutrition Survey II (2005–2007) [Max Rubner-Institut. Nationale Verzehrsstudie II. Ergebnisbericht, Teil 2: Die bundesweite Befragung zur Ernährung von Jugendlichen und Erwachsenen. p. 253. MRI, Karlsruhe, Germany (2008). Available at: https://www.openagrar.de/receive/bmelv_mods_00000135 (Accessed February 24, 2026) (109)]. Percentage of people (male (m), n = 7093; female (f), n = 8278) of different ages who do not reach the recommended vitamin B6 intake according to D-A-CH reference values [German Nutrition Society (DGE); Austrian Nutrition Society (ÖGE); Swiss Nutrition Society (SGE). Referenzwerte für die Nährstoffzufuhr [Dietary Reference Values], 2nd edition, 5th updated issue; German Nutrition Society: Bonn, Germany, 2019; ISBN 9783887492618.]. Mean values are represented as dotted lines. D-A-CH, Deutschland, Austria, Confoederatio Helvetica (eng. GSA, Germany, Switzerland, Austria DGE, Deutsche Gesellschaft für Ernährung; ÖGE, Österreichische Gesellschaft für Ernährung; SGE, Schweizerische Gesellschaft für Ernährung.).

Supplementary Figure 5 Results of the German National Nutrition Survey II (2005–2007) [Max Rubner-Institut. Nationale Verzehrsstudie II. Ergebnisbericht, Teil 2: Die bundesweite Befragung zur Ernährung von Jugendlichen und Erwachsenen. p. 254. MRI, Karlsruhe, Germany (2008). Available at: https://www.openagrar.de/receive/bmelv_mods_00000135 (Accessed February 24, 2026) (154)]. Percentage of people (male (m), n = 7093; female (f), n = 8278) of different ages who do not reach the recommended vitamin B9 (folate) intake according to D-A-CH reference values [German Nutrition Society (DGE); Austrian Nutrition Society (ÖGE); Swiss Nutrition Society (SGE). Referenzwerte für die Nährstoffzufuhr [Dietary Reference Values], 2nd edition, 5th updated issue; German Nutrition Society: Bonn, Germany, 2019; ISBN 9783887492618.]. Mean values are represented as dotted lines. D-A-CH, Deutschland, Austria, Confoederatio Helvetica (eng. GSA, Germany, Switzerland, Austria DGE, Deutsche Gesellschaft für Ernährung; ÖGE, Österreichische Gesellschaft für Ernährung; SGE, Schweizerische Gesellschaft für Ernährung.).

Supplementary Figure 6 Results of the German National Nutrition Survey II (2005–2007) [Max Rubner-Institut. Nationale Verzehrsstudie II. Ergebnisbericht, Teil 2: Die bundesweite Befragung zur Ernährung von Jugendlichen und Erwachsenen. p. 255. MRI, Karlsruhe, Germany (2008). Available at: https://www.openagrar.de/receive/bmelv_mods_00000135 (Accessed February 24, 2026) (172)]. Percentage of people (male (m), n = 7093; female (f), n = 8278) of different ages who do not reach the recommended vitamin B12 (cobalamin) intake according to D-A-CH reference values [German Nutrition Society (DGE); Austrian Nutrition Society (ÖGE); Swiss Nutrition Society (SGE). Referenzwerte für die Nährstoffzufuhr [Dietary Reference Values], 2nd edition, 5th updated issue; German Nutrition Society: Bonn, Germany, 2019; ISBN 9783887492618.]. Mean values are represented as dotted lines. D-A-CH, Deutschland, Austria, Confoederatio Helvetica (eng. GSA, Germany, Switzerland, Austria, DGE, Deutsche Gesellschaft für Ernährung; ÖGE, Österreichische Gesellschaft für Ernährung; SGE, Schweizerische Gesellschaft für Ernährung.).

Supplementary Figure 7 Results of the German National Nutrition Survey II (2005–2007) [Max Rubner-Institut. Nationale Verzehrsstudie II. Ergebnisbericht, Teil 2: Die bundesweite Befragung zur Ernährung von Jugendlichen und Erwachsenen. p. 256. MRI, Karlsruhe, Germany (2008). Available at: https://www.openagrar.de/receive/bmelv_mods_00000135 (Accessed February 24, 2026) (190)]. Percentage of people (male (m), n = 7093; female (f), n = 8278) of different ages who do not reach the recommended vitamin C (ascorbate) intake according to D-A-CH reference values [German Nutrition Society (DGE); Austrian Nutrition Society (ÖGE); Swiss Nutrition Society (SGE). Referenzwerte für die Nährstoffzufuhr [Dietary Reference Values], 2nd edition, 5th updated issue; German Nutrition Society: Bonn, Germany, 2019; ISBN 9783887492618.]. Mean values are represented as dotted lines. D-A-CH, Deutschland, Austria, Confoederatio Helvetica (eng. GSA, Germany, Switzerland, Austria). DGE, Deutsche Gesellschaft für Ernährung; ÖGE, Österreichische Gesellschaft für Ernährung; SGE, Schweizerische Gesellschaft für Ernährung.

## Glossary

5 MTHF: 5-methyl tetrahydrofolate
AGE: advanced glycation end product
Bcl-2: B-cell lymphoma 2
CoA: coenzyme A
COVID-19: coronavirus disease 2019
COX-2: cyclooxygenase-2
CRP: c-reactive protein
D-A-CH: Deutschland, Austria, Confoederatio Helvetica
DAMP: damage-associated molecular pattern
DFE: dietary folate equivalents
DGE: Deutsche Gesellschaft für Ernährung
DGE: Deutsche Gesellschaft für Ernährung (eng. German Nutrition Society)
DRI: Dietary Reference Intake
DTH: delayed-type hypersensitivity
FAD: flavin adenine dinucleotide
FMN: flavin mononucleotide
GABA: gamma-aminobutyric acid
GSA: Germany, Switzerland, Austria
ICAM: intracellular adhesion molecule
IFN-γ: interferon gamma
IL-6: interleukin-6
iNOS: inducible nitric oxide synthase
LPS: lipopolysaccharide
MAIT: mucosal-associated invariant T
MCP-1: monocyte chemoattractant protein-1
MR1: major histocompatibility complex class I-related protein 1
mTOR: mechanistic target of rapamycin
n/a: not available
NE: niacin equivalents
NF-kB: nuclear factor kappa-light-chain-enhancer of activated B cells
NIACR1: nicotinic acid receptor 1
NIH: National Institutes of Health
NLR: NOD-like receptor
NOD: nucleotide-binding oligomerization domain
NLRP3: NLR family pyrin domain containing 3
NO: nitric oxide
NRV: Nutrient Reference Value
NVS II: Nationale Verzehrstudie II (eng. National Nutrition Survey II)
PBMC: peripheral blood mononuclear cell
PGE2: prostaglandin E2
PLP: pyridoxal-5-phosphate
RCT: randomized controlled trial
RDA: Recommended Dietary Allowance
ROS: reactive oxygen species
SARS-CoV-2: severe acute respiratory syndrome coronavirus type 2
SP1/3: specificity proteins 1 and 3
TCA: tricarboxylic acid
THF: tetrahydrofolate
TNF-α: tumor necrosis factor α
TPP: thiamine pyrophosphate
Treg: regulatory T cell
UI: Tolerable Upper Intake Level
UL: Tolerable Upper Intake Level
WHO: World Health Organization
y/o: years old
