# Supplementary material for: Exploring the link between water-soluble vitamins and aging-associated immune system status
Source: Front Immunol. 2026 May 26;17:1771591. doi: 10.3389/fimmu.2026.1771591 (PMC13246420; doi:10.3389/fimmu.2026.1771591)
Supplement: Supplementary Figure 1 — Results of the German National Nutrition Survey II (2005–2007) [Max Rubner-Institut. Nationale Verzehrsstudie II. Ergebnisbericht, Teil 2: Die bundesweite Befragung zur Ernährung von Jugendlichen und Erwachsenen. p. 250. MRI, Karlsruhe, Germany (2008). Available at: https://www.openagrar.de/receive/bmelv_mods_00000135 (Accessed February 24, 2026) (46)]. Percentage of people [male (m), n = 7093; female (f), n = 8278] of different ages who do not reach the recommended vitamin B1 intake according to D-A-CH reference values [German Nutrition Society (DGE); Austrian Nutrition Society (ÖGE); Swiss Nutrition Society (SGE). Referenzwerte für die Nährstoffzufuhr [Dietary Reference Values], 2nd edition, 5th updated issue; German Nutrition Society: Bonn, Germany, 2019; ISBN 9783887492618.]. Mean values are represented as dotted lines. D-A-CH, Deutschland, Austria, Confoederatio Helvetica (eng. GSA, Germany, Switzerland, Austria) DGE, Deutsche Gesellschaft für Ernährung; ÖGE, Österreichische Gesellschaft für Ernährung; SGE, Schweizerische Gesellschaft für Ernährung.. [file DataSheet1.pdf]

Supplementary Material

1 Supplementary Figures and Tables

1.1 Supplementary Tables

Supplementary Table 1: Clinical and pre-clinical studies on water-soluble vitamins by study designs

Vitamin	Study Type	Reference
Thiamine	In vitro, in vivo (mice)	Hirata et al. (2020)
	In vivo (rats)	Pletsityi (1979)
	Observational/ cross-sectional ex vivo & biochemical in vitro study	Gangolf et al. (2010)
	In vitro	Bozic et al. (2015)
	In vitro	Shoeb und Ramana (2012)
	In vivo (sheep), ex vivo	Olkowski et al. (1990)
Riboflavin	In vitro	Mazur-Bialy et al. (2015)<br>Mazur-Bialy und Pocheć (2016)<br>Mazur-Bialy und Pocheć (2017)
	In vitro	Dey und Bishayi (2016)
	in vitro & structural biology studies	Kjer-Nielsen et al. (2012)<br>Corbett et al. (2014)
Niacin	In vitro	Digby et al. (2012)
	In vitro	Chai et al. (2013)
	In vitro/ ex vivo	La Montserrat-de Paz et al. (2017)
	In vitro, in vivo (mice)	Singh et al. (2014)

	In vitro, in vivo (guinea pigs)	Si et al. (2014)
Pantothenic acid	In vivo (mice)	He et al. (2018)
	In vivo (mice), in vitro	St Paul et al. (2021)
	Prospective cohort study; community-based longitudinal study	Jung et al. (2017)
	In vivo (rats)	Karadag et al. (2015)
	In vivo (mice)	Li-Mei et al. (2016)
	In vivo (mice), in vitro	Berruyer et al. (2006)
	Translational research study with multimodal design (in vivo (mice), ex vivo, in silico)	Millet et al. (2023)
Pyridoxine	Double-blind, placebo-controlled clinical trial	Talbott et al. (1987)
	Controlled, experimental metabolic depletion-repletion study	Meydani et al. (1991)
	Observational/ cross-sectional analysis of a prospective cohort	Sakakeeny et al. (2012)
	In vivo (mice)	Qian et al. (2017)
	In vivo (grass carp)	Zheng et al. (2017)
	In vivo (mice), in vitro	Zhang et al. (2016)
	Prospective, multicenter, double-blind, randomized placebo-controlled trial	Naurath et al. (1995)
Biotin	In vivo (rats)	Rabin (1983)
	In vivo (mice)	Kuroishi et al. (2009)
	In vitro	Agrawal et al. (2016)
	In vivo (mice), In vitro	Elahi et al. (2018)
Folate	In vivo (rats)	Partearroyo et al. (2013)

	Cross-sectional observational study	Troen et al. (2006)
	In vivo (rats)	Field et al. (2006)
	Randomized, controlled, longitudinal clinical intervention	Bunout et al. (2004)
	In silico	Sheybani et al. (2020)
	In silico	Kumar et al. (2021)
	Prospective, multicenter, double-blind, randomized placebo-controlled trial	Naurath et al. (1995)
Cobalamin	Prospective, multicenter, double-blind, randomized placebo-controlled trial	Naurath et al. (1995)
	In vivo (rats)	Partearroyo et al. (2013)
	Randomized, controlled, longitudinal clinical intervention	Bunout et al. (2004)
	Controlled, prospective cohort study	Fata et al. (1996)
Vitamin C	Ex vivo	Härtel et al. (2007)
	Randomized, double-blind, placebo-controlled trial	Johnston et al. (2014)
	Placebo-controlled, randomized, longitudinal intervention trial with ex vivo analysis	Jeng et al. (1996)
	In vitro	Huijskens et al. (2015)
	Double-blind, placebo-controlled, clinical intervention trial	Kennes et al. (1983)
	Randomized, double-blind, placebo-controlled trial	Żychowska et al. (2021)
	Prospective, clinical intervention trial	Andrews et al. (1969)

References

Berruyer, Carole; Pouyet, Laurent; Millet, Virginie; Martin, Florent M.; LeGoffic, Aude; Canonici, Alexandra et al. (2006): Vanin-1 licenses inflammatory mediator production by gut epithelial cells and controls colitis by antagonizing peroxisome proliferator-activated receptor gamma activity. In: *The Journal of experimental medicine* 203 (13), S. 2817–2827. DOI: 10.1084/jem.20061640.

Bozic, Iva; Savic, Danijela; Laketa, Danijela; Bjelobaba, Ivana; Milenkovic, Ivan; Pekovic, Sanja et al. (2015): Benfotiamine attenuates inflammatory response in LPS stimulated BV-2 microglia. In: *PloS one* 10 (2), e0118372. DOI: 10.1371/journal.pone.0118372.

Chai, Joshua T.; Digby, Janet E.; Ruparelia, Neil; Jefferson, Andrew; Handa, Ashok; Choudhury, Robin P. (2013): Nicotinic acid receptor GPR109A is down-regulated in human macrophage-derived foam cells. In: *PloS one* 8 (5), e62934. DOI: 10.1371/journal.pone.0062934.

Corbett, Alexandra J.; Eckle, Sidonia B. G.; Birkinshaw, Richard W.; Liu, Ligong; Patel, Onisha; Mahony, Jennifer et al. (2014): T-cell activation by transitory neo-antigens derived from distinct microbial pathways. In: *Nature* 509 (7500), S. 361–365. DOI: 10.1038/nature13160.

Dey, Somrita; Bishayi, Biswadev (2016): Riboflavin along with antibiotics balances reactive oxygen species and inflammatory cytokines and controls Staphylococcus aureus infection by boosting murine macrophage function and regulates inflammation. In: *Journal of inflammation (London, England)* 13, S. 36. DOI: 10.1186/s12950-016-0145-0.

Digby, Janet E.; Martinez, Fernando; Jefferson, Andrew; Ruparelia, Neil; Chai, Joshua; Wamil, Malgorzata et al. (2012): Anti-inflammatory effects of nicotinic acid in human monocytes are mediated by GPR109A dependent mechanisms. In: *Arteriosclerosis, thrombosis, and vascular biology* 32 (3), S. 669–676. DOI: 10.1161/ATVBAHA.111.241836.

Gangolf, Marjorie; Czerniecki, Jan; Radermecker, Marc; Detry, Olivier; Nisolle, Michelle; Jouan, Caroline et al. (2010): Thiamine status in humans and content of phosphorylated thiamine derivatives in biopsies and cultured cells. In: *PloS one* 5 (10), e13616. DOI: 10.1371/journal.pone.0013616.

He, Wenting; Hu, Shengfeng; Du, Xialin; Wen, Qian; Zhong, Xiao-Ping; Zhou, Xinying et al. (2018): Vitamin B5 Reduces Bacterial Growth via Regulating Innate Immunity and Adaptive Immunity in Mice Infected with Mycobacterium tuberculosis. In: *Frontiers in immunology* 9, S. 365. DOI: 10.3389/fimmu.2018.00365.

Hirata, So-Ichiro; Sawane, Kento; Adachi, Jun; Isoyama, Junko; Sugiura, Yuki; Matsunaga, Ayu et al. (2020): Vitamin B1 Supports the Differentiation of T Cells through TGF-β Superfamily Production in Thymic Stromal Cells. In: *iScience* 23 (9), S. 101426. DOI: 10.1016/j.isci.2020.101426.

Jung, S.; Kim, M. K.; Choi, B. Y. (2017): The long-term relationship between dietary pantothenic acid (vitamin B5) intake and C-reactive protein concentration in adults aged 40 years and older. In: *Nutrition, metabolism, and cardiovascular diseases : NMCD* 27 (9), S. 806–816. DOI: 10.1016/j.numecd.2017.05.008.

Karadag, Ahmet; Ozdemir, Ramazan; Kurt, Ahmet; Parlakpinar, Hakan; Polat, Alaadin; Vardi, Nigar et al. (2015): Protective effects of dexpanthenol in an experimental model of necrotizing enterocolitis. In: *Journal of Pediatric Surgery* 50 (7), S. 1119–1124. DOI: 10.1016/j.jpedsurg.2014.10.053.

Kjer-Nielsen, Lars; Patel, Onisha; Corbett, Alexandra J.; Le Nours, Jérôme; Meehan, Bronwyn; Liu, Ligong et al. (2012): MR1 presents microbial vitamin B metabolites to MAIT cells. In: *Nature* 491 (7426), S. 717–723. DOI: 10.1038/nature11605.

La Montserrat-de Paz, Sergio; Naranjo, M. Carmen; Lopez, Sergio; Abia, Rocio; Muriana, Francisco J. Garcia; Bermudez, Beatriz (2017): Niacin and its metabolites as master regulators of

macrophage activation. In: *The Journal of nutritional biochemistry* 39, S. 40–47. DOI: 10.1016/j.jnutbio.2016.09.008.

Li-Mei, Wan; Jie, Tan; Shan-He, Wan; Dong-Mei, Meng; Peng-Jiu, Yu (2016): Anti-inflammatory and Anti-oxidative Effects of Dexpanthenol on Lipopolysaccharide Induced Acute Lung Injury in Mice. In: *Inflammation* 39 (5), S. 1757–1763. DOI: 10.1007/s10753-016-0410-7.

Mazur-Bialy, A. I.; Pochec, E.; Plytycz, B. (2015): Immunomodulatory effect of riboflavin deficiency and enrichment - reversible pathological response versus silencing of inflammatory activation. In: *Journal of physiology and pharmacology : an official journal of the Polish Physiological Society* 66 (6), S. 793–802.

Mazur-Bialy, Agnieszka Irena; Pocheć, Ewa (2016): Riboflavin Reduces Pro-Inflammatory Activation of Adipocyte-Macrophage Co-culture. Potential Application of Vitamin B2 Enrichment for Attenuation of Insulin Resistance and Metabolic Syndrome Development. In: *Molecules (Basel, Switzerland)* 21 (12). DOI: 10.3390/molecules21121724.

Mazur-Bialy, Agnieszka Irena; Pocheć, Ewa (2017): Vitamin B2 deficiency enhances the pro-inflammatory activity of adipocyte, consequences for insulin resistance and metabolic syndrome development. In: *Life sciences* 178, S. 9–16. DOI: 10.1016/j.lfs.2017.04.010.

Meydani, S. N.; Ribaya-Mercado, J. D.; Russell, R. M.; Sahyoun, N.; Morrow, F. D.; Gershoff, S. N. (1991): Vitamin B-6 deficiency impairs interleukin 2 production and lymphocyte proliferation in elderly adults. In: *The American journal of clinical nutrition* 53 (5), S. 1275–1280. DOI: 10.1093/ajcn/53.5.1275.

Millet, Virginie; Gensollen, Thomas; Maltese, Michael; Serrero, Melanie; Lesavre, Nathalie; Bourges, Christophe et al. (2023): Harnessing the Vnn1 pantetheinase pathway boosts short chain fatty acids production and mucosal protection in colitis. In: *Gut* 72 (6), S. 1115–1128. DOI: 10.1136/gutjnl-2021-325792.

Olkowski, A. A.; Gooneratne, S. R.; Christensen, D. A. (1990): Effects of diets of high sulphur content and varied concentrations of copper, molybdenum and thiamine on in vitro phagocytic and candidacidal activity of neutrophils in sheep. In: *Research in veterinary science* 48 (1), S. 82–86.

Pletsityi, A. D. (1979): Changes in activity of some mechanisms of specific and nonspecific immunity in vitamin B1 deficiency. In: *Bull Exp Biol Med* 88 (1), S. 741–743. DOI: 10.1007/BF00804782.

Qian, Bingjun; Shen, Shanqi; Zhang, Jianhua; Jing, Pu (2017): Effects of Vitamin B6 Deficiency on the Composition and Functional Potential of T Cell Populations. In: *Journal of immunology research* 2017, S. 2197975. DOI: 10.1155/2017/2197975.

Sakakeeny, Lydia; Roubenoff, Ronenn; Obin, Martin; Fontes, Joao D.; Benjamin, Emelia J.; Bujanover, Yoram et al. (2012): Plasma Pyridoxal-5-Phosphate Is Inversely Associated with Systemic Markers of Inflammation in a Population of U.S. Adults,. In: *The Journal of nutrition* 142 (7), S. 1280–1285. DOI: 10.3945/jn.111.153056.

Shoeb, Mohammad; Ramana, Kota V. (2012): Anti-inflammatory effects of benfotiamine are mediated through the regulation of the arachidonic acid pathway in macrophages. In: *Free radical biology & medicine* 52 (1), S. 182–190. DOI: 10.1016/j.freeradbiomed.2011.10.444.

Si, Yanhong; Zhang, Ying; Zhao, Jilong; Guo, Shoudong; Zhai, Lei; Yao, Shutong et al. (2014): Niacin inhibits vascular inflammation via downregulating nuclear transcription factor-κB signaling pathway. In: *Mediators of inflammation* 2014, S. 263786. DOI: 10.1155/2014/263786.

Singh, Nagendra; Gurav, Ashish; Sivaprakasam, Sathish; Brady, Evan; Padia, Ravi; Shi, Huidong et al. (2014): Activation of Gpr109a, receptor for niacin and the commensal metabolite butyrate, suppresses colonic inflammation and carcinogenesis. In: *Immunity* 40 (1), S. 128–139. DOI: 10.1016/j.immuni.2013.12.007.

St Paul, Michael; Saibil, Samuel D.; Han, SeongJun; Israni-Winger, Kavita; Lien, Scott C.; Laister, Rob C. et al. (2021): Coenzyme A fuels T cell anti-tumor immunity. In: *Cell metabolism* 33 (12), 2415-2427.e6. DOI: 10.1016/j.cmet.2021.11.010.

Talbott, M. C.; Miller, L. T.; Kerkvliet, N. I. (1987): Pyridoxine supplementation: effect on lymphocyte responses in elderly persons. In: *The American journal of clinical nutrition* 46 (4), S. 659–664. DOI: 10.1093/ajcn/46.4.659.

Zhang, Peipei; Tsuchiya, Kohsuke; Kinoshita, Takeshi; Kushiyama, Hiroko; Suidasari, Sofya; Hatakeyama, Mizuki et al. (2016): Vitamin B6 Prevents IL-1β Protein Production by Inhibiting NLRP3 Inflammasome Activation. In: *The Journal of biological chemistry* 291 (47), S. 24517–24527. DOI: 10.1074/jbc.M116.743815.

Zheng, Xin; Feng, Lin; Jiang, Wei-Dan; Wu, Pei; Liu, Yang; Jiang, Jun et al. (2017): Dietary pyridoxine deficiency reduced growth performance and impaired intestinal immune function associated with TOR and NF-κB signalling of young grass carp (Ctenopharyngodon idella). In: *Fish & Shellfish Immunology* 70, S. 682–700. DOI: 10.1016/j.fsi.2017.09.055.

1.2 Supplementary Figures

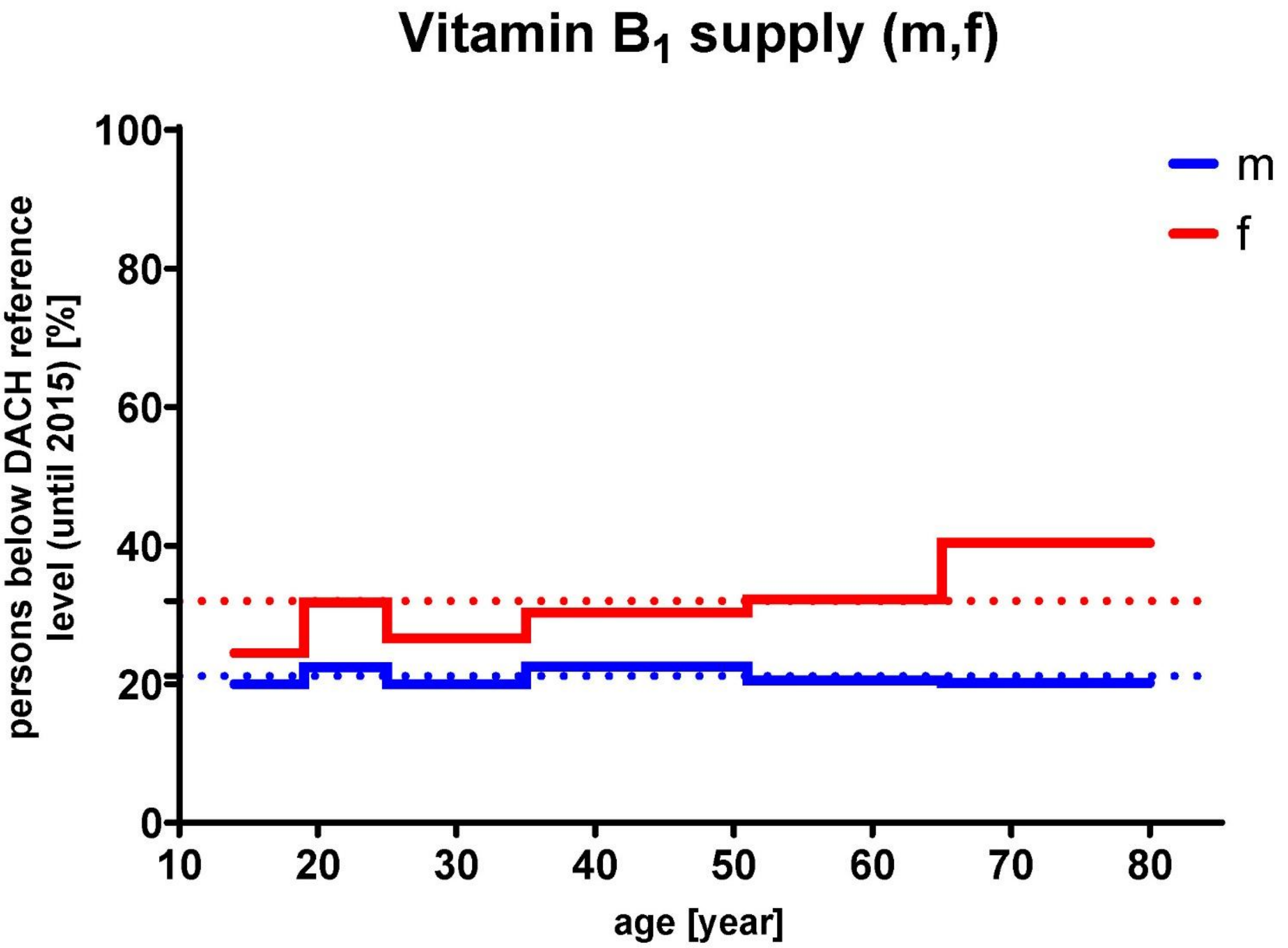

Supplementary Figure 1: Results of the German National Nutrition Survey II (2005–2007) (Max Rubner-Institut. Nationale Verzehrsstudie II. Ergebnisbericht, Teil 2: Die bundesweite Befragung zur Ernährung von Jugendlichen und Erwachsenen. p. 250. MRI, Karlsruhe, Germany (2008). Available at: https://www.openagrar.de/receive/bmelv_mods_00000135 (accessed February 24, 2026). Percentage of people (male (m), n = 7093; female (f), n = 8278) of different ages who do not reach the recommended vitamin B1 intake according to D-A-CH reference values (German Nutrition Society (DGE); Austrian Nutrition Society (ÖGE); Swiss Nutrition Society (SGE). Referenzwerte für die Nährstoffzufuhr [Dietary Reference Values], 2nd edition, 5th updated issue; German Nutrition Society: Bonn, Germany, 2019; ISBN 9783887492618). Mean values are represented as dotted lines. D-A-CH, Deutschland, Austria, Confoederatio Helvetica (eng. GSA, Germany, Switzerland, Austria); DGE, Deutsche Gesellschaft für Ernährung; ÖGE, Österreichische Gesellschaft für Ernährung; SGE, Schweizerische Gesellschaft für Ernährung.

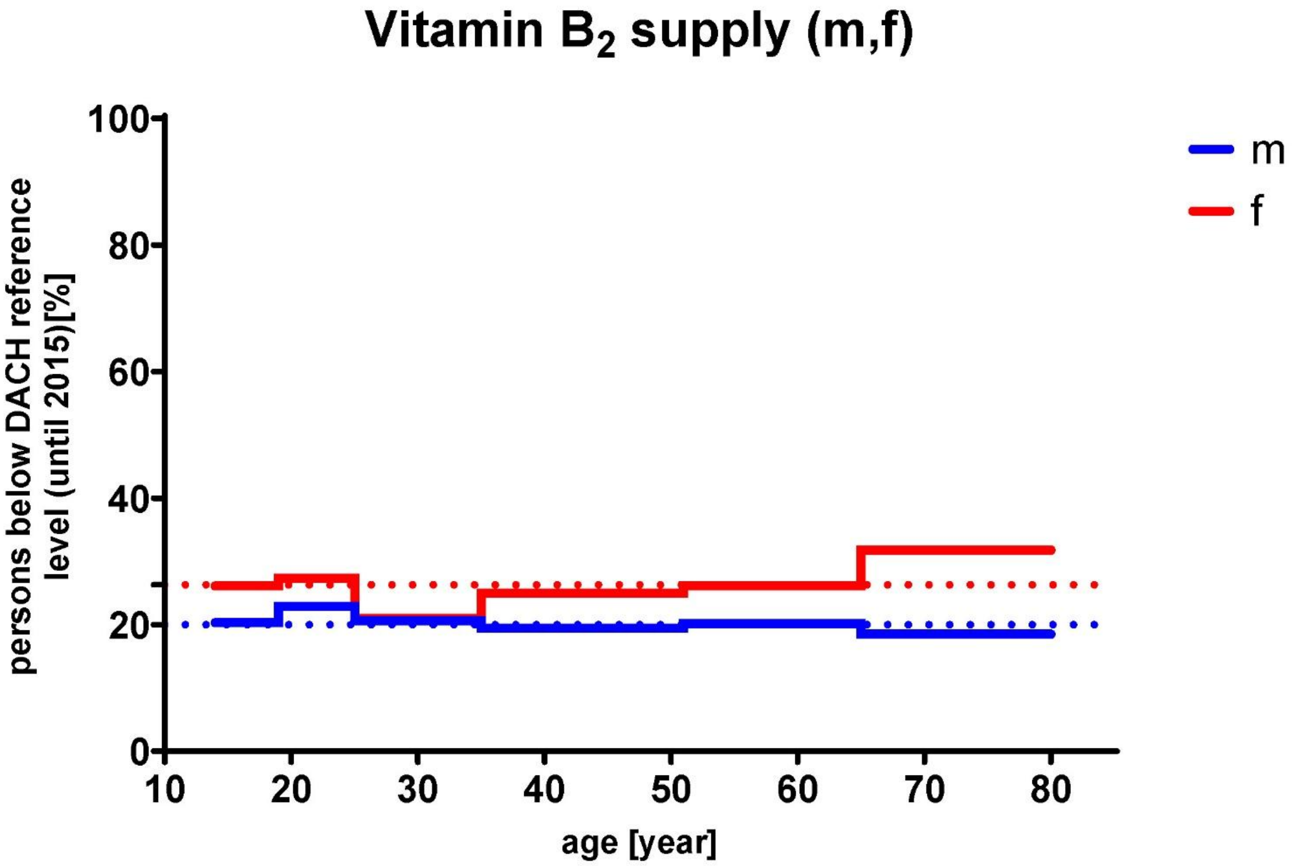

Supplementary Figure 2: Results of the German National Nutrition Survey II (2005–2007) (Max Rubner-Institut. Nationale Verzehrsstudie II. Ergebnisbericht, Teil 2: Die bundesweite Befragung zur Ernährung von Jugendlichen und Erwachsenen. p. 251. MRI, Karlsruhe, Germany (2008). Available at: https://www.openagrar.de/receive/bmelv_mods_00000135 (accessed February 24, 2026). Percentage of people (male (m), n = 7093; female (f), n = 8278) of different ages who do not reach the recommended vitamin B2 intake according to D-A-CH reference values (German Nutrition Society (DGE); Austrian Nutrition Society (ÖGE); Swiss Nutrition Society (SGE). Referenzwerte für die Nährstoffzufuhr [Dietary Reference Values], 2nd edition, 5th updated issue; German Nutrition Society: Bonn, Germany, 2019; ISBN 9783887492618). Mean values are represented as dotted lines. D-A-CH, Deutschland, Austria, Confoederatio Helvetica (eng. GSA, Germany, Switzerland, Austria); DGE, Deutsche Gesellschaft für Ernährung; ÖGE, Österreichische Gesellschaft für Ernährung; SGE, Schweizerische Gesellschaft für Ernährung.

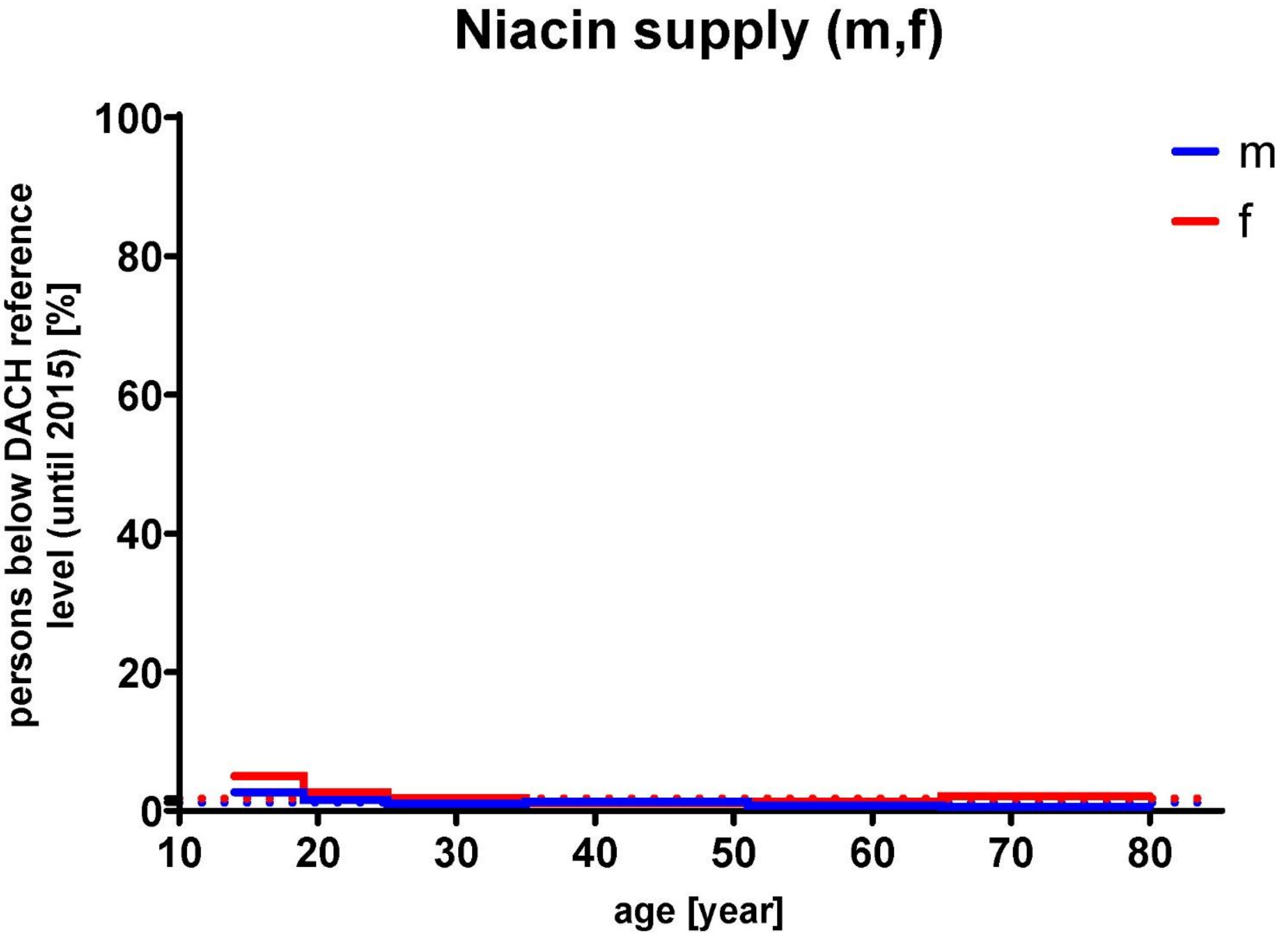

Supplementary Figure 3: Results of the German National Nutrition Survey II (2005–2007) (Max Rubner-Institut. Nationale Verzehrsstudie II. Ergebnisbericht, Teil 2: Die bundesweite Befragung zur Ernährung von Jugendlichen und Erwachsenen. p. 252. MRI, Karlsruhe, Germany (2008). Available at: https://www.openagrar.de/receive/bmelv_mods_00000135 (accessed February 24, 2026). Percentage of people (male (m), n = 7093; female (f), n = 8278) of different ages who do not reach the recommended vitamin B3 (niacin) intake according to D-A-CH reference values (German Nutrition Society (DGE); Austrian Nutrition Society (ÖGE); Swiss Nutrition Society (SGE). Referenzwerte für die Nährstoffzufuhr [Dietary Reference Values], 2nd edition, 5th updated issue; German Nutrition Society: Bonn, Germany, 2019; ISBN 9783887492618). Mean values are represented as dotted lines. D-A-CH, Deutschland, Austria, Confoederatio Helvetica (eng. GSA, Germany, Switzerland, Austria); DGE, Deutsche Gesellschaft für Ernährung; ÖGE, Österreichische Gesellschaft für Ernährung; SGE, Schweizerische Gesellschaft für Ernährung.

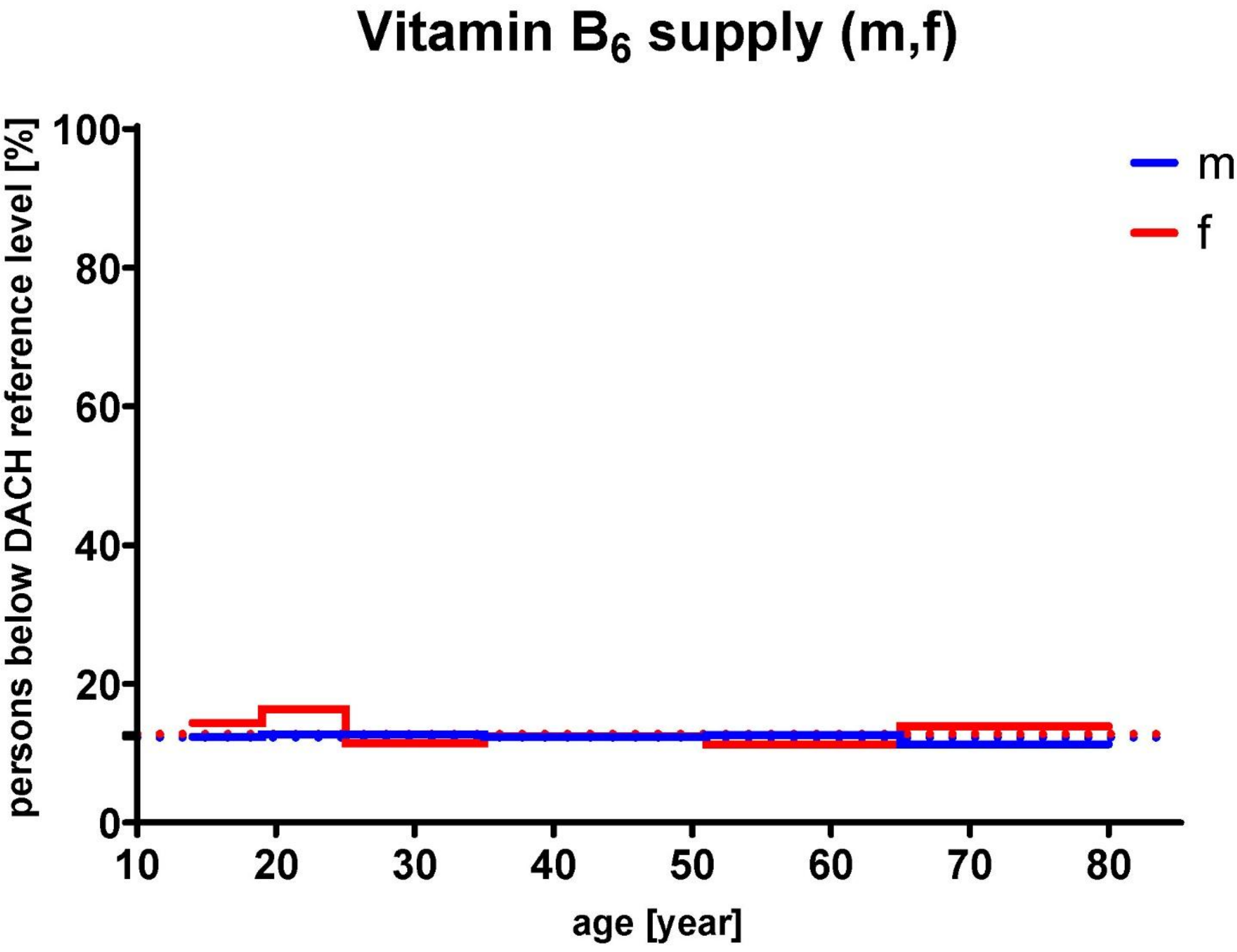

Supplementary Figure 4: Results of the German National Nutrition Survey II (2005–2007) (Max Rubner-Institut. Nationale Verzehrsstudie II. Ergebnisbericht, Teil 2: Die bundesweite Befragung zur Ernährung von Jugendlichen und Erwachsenen. p. 253. MRI, Karlsruhe, Germany (2008). Available at: https://www.openagrar.de/receive/bmelv_mods_00000135 (accessed February 24, 2026). Percentage of people (male (m), n = 7093; female (f), n = 8278) of different ages who do not reach the recommended vitamin B6 intake according to D-A-CH reference values (German Nutrition Society (DGE); Austrian Nutrition Society (ÖGE); Swiss Nutrition Society (SGE). Referenzwerte für die Nährstoffzufuhr [Dietary Reference Values], 2nd edition, 5th updated issue; German Nutrition Society: Bonn, Germany, 2019; ISBN 9783887492618). Mean values are represented as dotted lines. D-A-CH, Deutschland, Austria, Confoederatio Helvetica (eng. GSA, Germany, Switzerland, Austria); DGE, Deutsche Gesellschaft für Ernährung; ÖGE, Österreichische Gesellschaft für Ernährung; SGE, Schweizerische Gesellschaft für Ernährung.

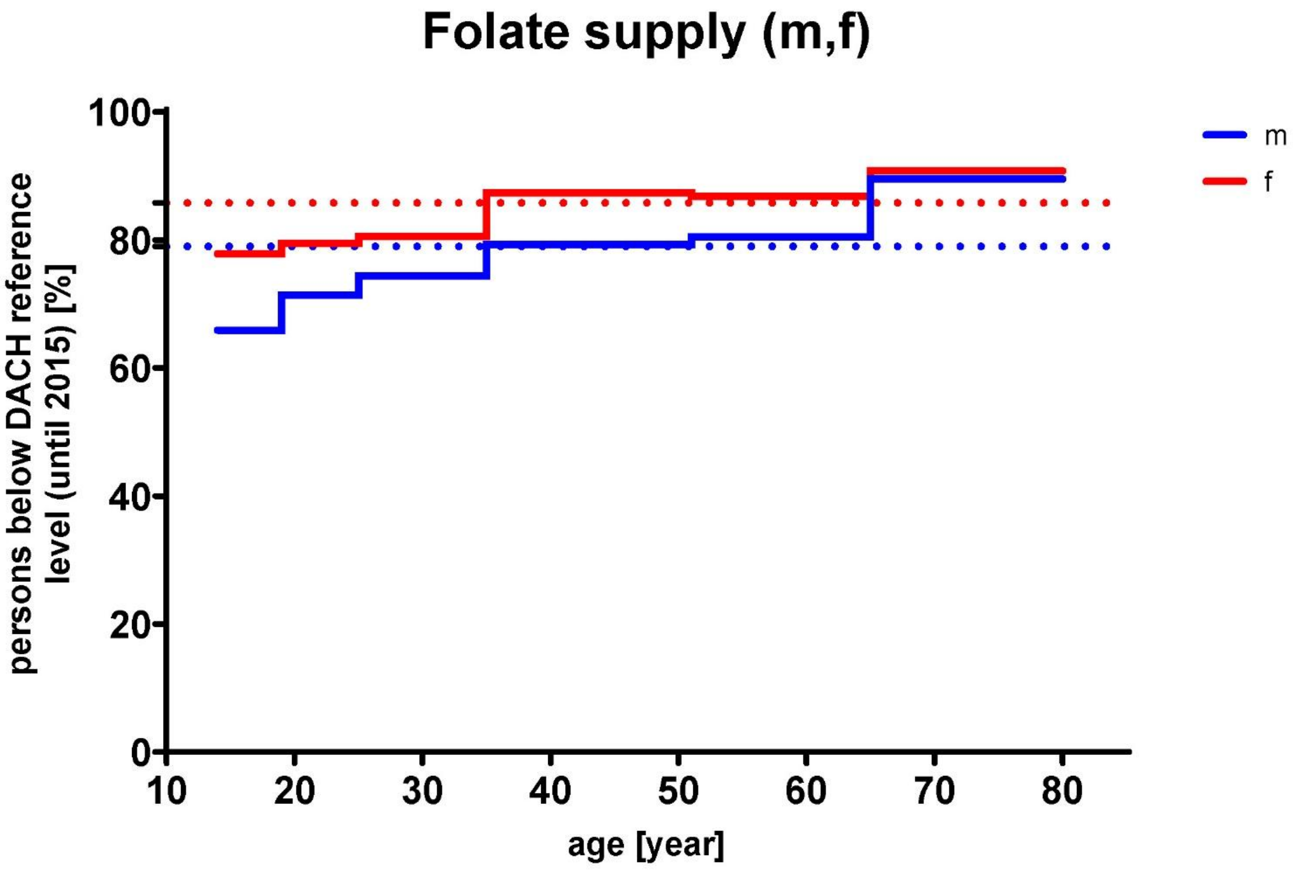

Supplementary Figure 5: Results of the German National Nutrition Survey II (2005–2007) (Max Rubner-Institut. Nationale Verzehrsstudie II. Ergebnisbericht, Teil 2: Die bundesweite Befragung zur Ernährung von Jugendlichen und Erwachsenen. p. 254. MRI, Karlsruhe, Germany (2008). Available at: https://www.openagrar.de/receive/bmelv_mods_00000135 (accessed February 24, 2026). Percentage of people (male (m), n = 7093; female (f), n = 8278) of different ages who do not reach the recommended vitamin B9 (folate) intake according to D-A-CH reference values (German Nutrition Society (DGE); Austrian Nutrition Society (ÖGE); Swiss Nutrition Society (SGE). Referenzwerte für die Nährstoffzufuhr [Dietary Reference Values], 2nd edition, 5th updated issue; German Nutrition Society: Bonn, Germany, 2019; ISBN 9783887492618). Mean values are represented as dotted lines. D-A-CH, Deutschland, Austria, Confoederatio Helvetica (eng. GSA, Germany, Switzerland, Austria); DGE, Deutsche Gesellschaft für Ernährung; ÖGE, Österreichische Gesellschaft für Ernährung; SGE, Schweizerische Gesellschaft für Ernährung.

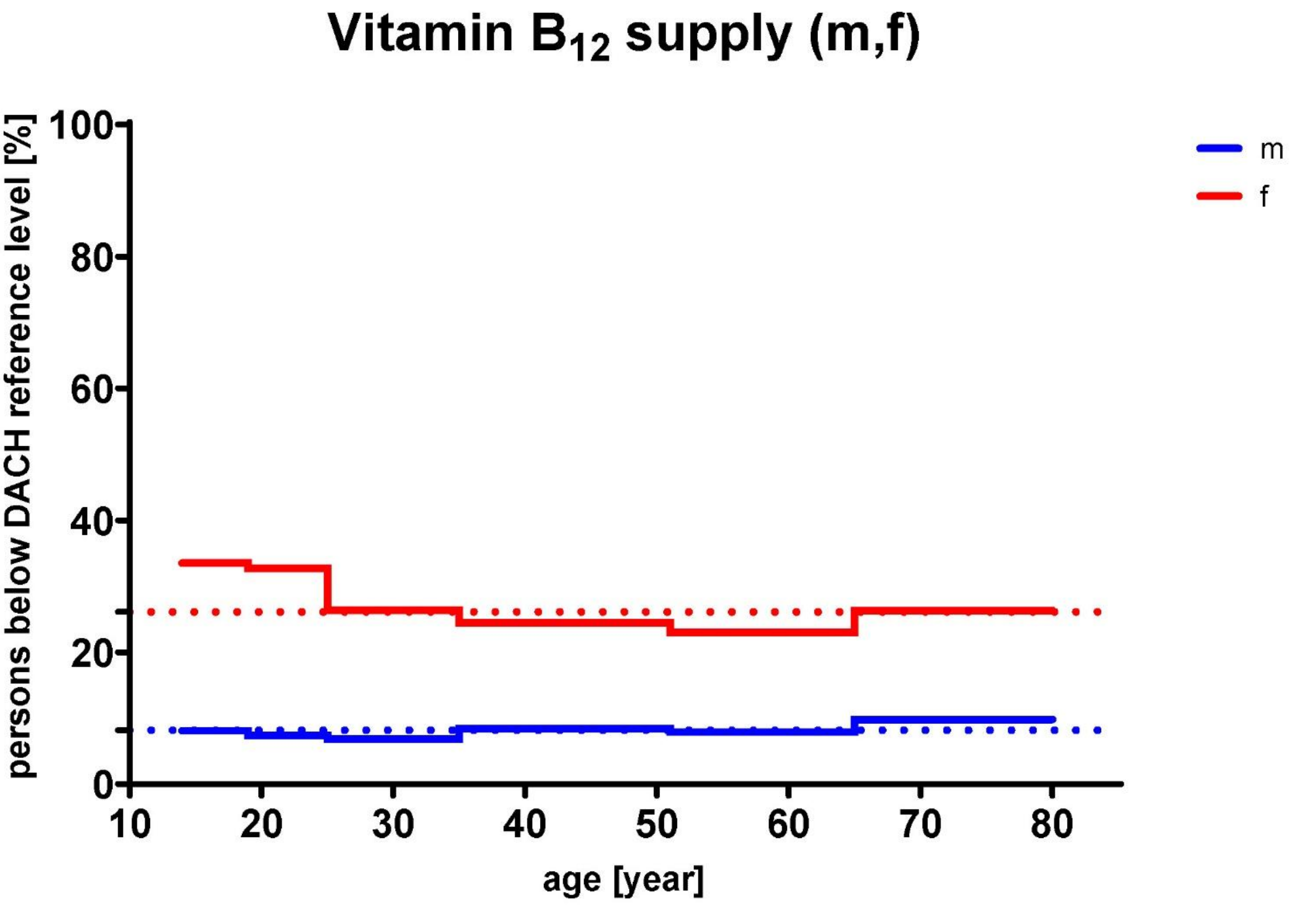

Supplementary Figure 6: Results of the German National Nutrition Survey II (2005–2007) (Max Rubner-Institut. Nationale Verzehrsstudie II. Ergebnisbericht, Teil 2: Die bundesweite Befragung zur Ernährung von Jugendlichen und Erwachsenen. p. 255. MRI, Karlsruhe, Germany (2008). Available at: https://www.openagrar.de/receive/bmelv_mods_00000135 (accessed February 24, 2026). Percentage of people (male (m), n = 7093; female (f), n = 8278) of different ages who do not reach the recommended vitamin B12 (cobalamin) intake according to D-A-CH reference values (German Nutrition Society (DGE); Austrian Nutrition Society (ÖGE); Swiss Nutrition Society (SGE). Referenzwerte für die Nährstoffzufuhr [Dietary Reference Values], 2nd edition, 5th updated issue; German Nutrition Society: Bonn, Germany, 2019; ISBN 9783887492618). Mean values are represented as dotted lines. D-A-CH, Deutschland, Austria, Confoederatio Helvetica (eng. GSA, Germany, Switzerland, Austria); DGE, Deutsche Gesellschaft für Ernährung; ÖGE, Österreichische Gesellschaft für Ernährung; SGE, Schweizerische Gesellschaft für Ernährung.

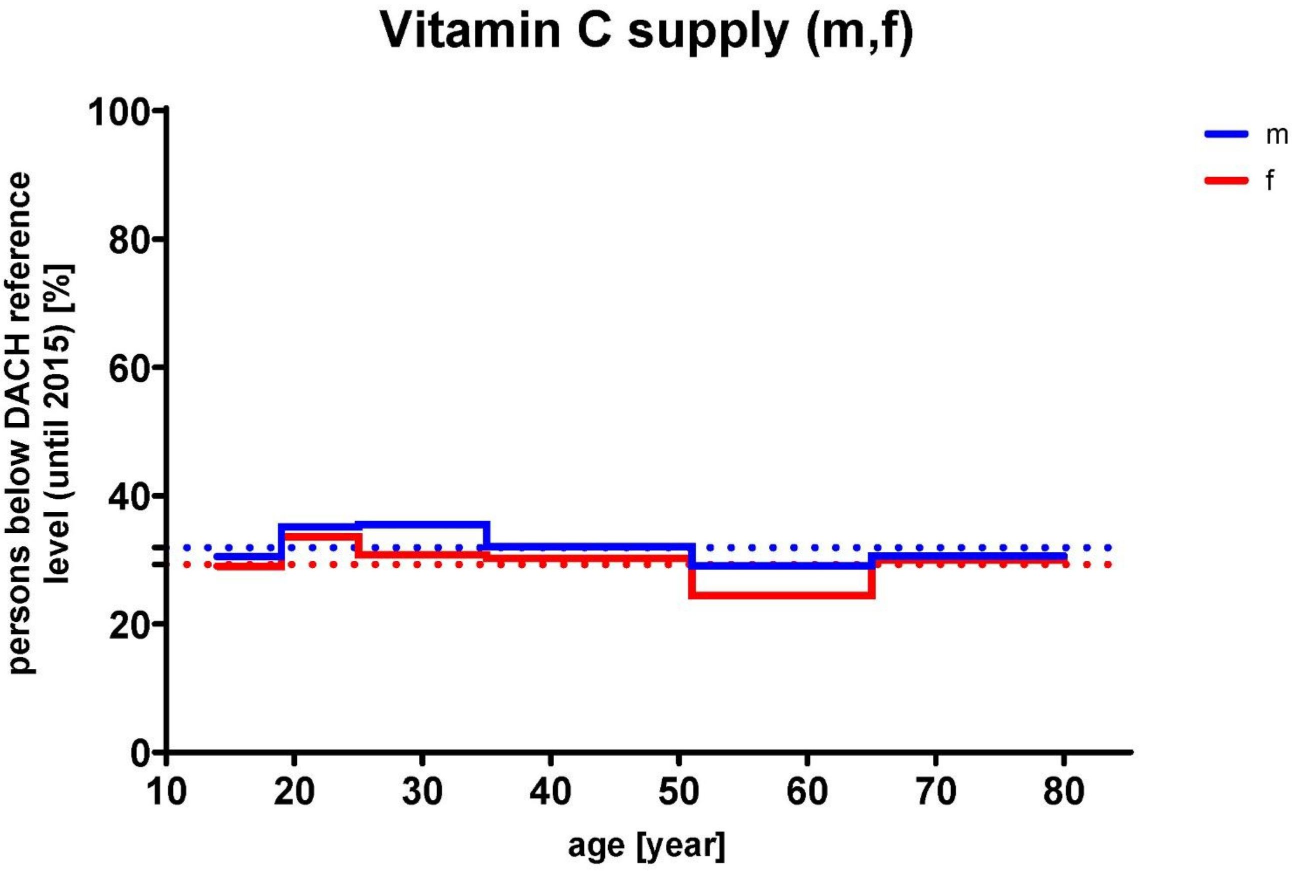

Supplementary Figure 7: Results of the German National Nutrition Survey II (2005–2007) (Max Rubner-Institut. Nationale Verzehrsstudie II. Ergebnisbericht, Teil 2: Die bundesweite Befragung zur Ernährung von Jugendlichen und Erwachsenen. p. 256. MRI, Karlsruhe, Germany (2008). Available at: https://www.openagrar.de/receive/bmelv_mods_00000135 (accessed February 24, 2026). Percentage of people (male (m), n = 7093; female (f), n = 8278) of different ages who do not reach the recommended vitamin C (ascorbate) intake according to D-A-CH reference values (German Nutrition Society (DGE); Austrian Nutrition Society (ÖGE); Swiss Nutrition Society (SGE). Referenzwerte für die Nährstoffzufuhr [Dietary Reference Values], 2nd edition, 5th updated issue; German Nutrition Society: Bonn, Germany, 2019; ISBN 9783887492618). Mean values are represented as dotted lines. D-A-CH, Deutschland, Austria, Confoederatio Helvetica (eng. GSA, Germany, Switzerland, Austria); DGE, Deutsche Gesellschaft für Ernährung; ÖGE, Österreichische Gesellschaft für Ernährung; SGE, Schweizerische Gesellschaft für Ernährung.
